# Toothbrush microbiomes feature a meeting ground for human oral and environmental microbiota

**DOI:** 10.1186/s40168-020-00983-x

**Published:** 2021-01-31

**Authors:** Ryan A. Blaustein, Lisa-Marie Michelitsch, Adam J. Glawe, Hansung Lee, Stefanie Huttelmaier, Nancy Hellgeth, Sarah Ben Maamar, Erica M. Hartmann

**Affiliations:** 1 Department of Civil and Environmental Engineering, Northwestern University, Evanston, IL USA; 2 National Human Genome Research Institute, National Institutes of Health, Bethesda, MD USA; 3 Institute of Environmental Biotechnology, Graz University of Technology, Graz, Austria

**Keywords:** Toothbrush, Oral microbiome, Built environment, Metagenomics, Antimicrobial resistance

## Abstract

**Background:**

While indoor microbiomes impact our health and well-being, much remains unknown about taxonomic and functional transitions that occur in human-derived microbial communities once they are transferred away from human hosts. Toothbrushes are a model to investigate the potential response of oral-derived microbiota to conditions of the built environment. Here, we characterize metagenomes of toothbrushes from 34 subjects to define the toothbrush microbiome and resistome and possible influential factors.

**Results:**

Toothbrush microbiomes often comprised a dominant subset of human oral taxa and less abundant or site-specific environmental strains. Although toothbrushes contained lower taxonomic diversity than oral-associated counterparts (determined by comparison with the *Human Microbiome Project*), they had relatively broader antimicrobial resistance gene (ARG) profiles. Toothbrush resistomes were enriched with a variety of ARGs, notably those conferring multidrug efflux and putative resistance to triclosan, which were primarily attributable to versatile environmental taxa. Toothbrush microbial communities and resistomes correlated with a variety of factors linked to personal health, dental hygiene, and bathroom features.

**Conclusions:**

Selective pressures in the built environment may shape the dynamic mixture of human (primarily oral-associated) and environmental microbiota that encounter each other on toothbrushes. Harboring a microbial diversity and resistome distinct from human-associated counterparts suggests toothbrushes could potentially serve as a reservoir that may enable the transfer of ARGs.

Video abstract

**Supplementary Information:**

The online version contains supplementary material available at 10.1186/s40168-020-00983-x.

## Background

Interactions between the human microbiome and surrounding environment influence microbial diversity central to our health and well-being [1, 2]. Microorganisms are transferred to and from hosts through direct contact (e.g., with surfaces) as well as via passive transmission (e.g., shedding of particles, aerosols). Accordingly, microbial assemblages that emerge in indoor spaces reflect human activity [3–5]. Putative selective pressures within the built environment (e.g., desiccation, limited resource availability, chemical residues) impact potential microbial adaptations and community dynamics [1, 3–8]. Since the human-associated microbiota in such indoor microbiome studies disproportionately involves the fraction derived from skin (i.e., microbial communities on surfaces, doorknobs, sinks, dust), the question of what happens to those from other body sites (e.g., the oral microbiome) when they find themselves in a hostile non-host environment is largely unexplored.

Toothbrushes are a unique component of the built environment that comes in contact with our bodies (i.e., mouth, hands) and water on a daily basis. Microenvironments on toothbrushes exhibit periodic wetting, variations in temperature, and differences in physical surfaces that may impact the ability of microbiota to colonize. Used toothbrushes are known to harbor a combination of putatively human-derived and environmental microorganisms, such as members of *Streptococcus*, *Staphylococcus*, *Pseudomonas*, *Poryphromonas*, *Parvimonas*, *Lactobacillus*, *Klebsiella*, *Fusobacterium*, *Escherichia*, and *Enterococcus* [9–12]. However, since these assessments were largely limited to culture-dependent monitoring for contamination, much remains unknown about microbial community structure and function on toothbrushes.

Mixed microbial communities that emerge on toothbrushes may be shaped by a variety of factors, particularly those that influence potential source microbiomes. For example, the human oral microbiome is associated with age, dental hygiene, and clinical health (e.g., dental caries, periodontal disease, and even oral cancer) [13–17]. As toothbrushes appear as a reservoir for both human-associated and environmental taxa, their microbial diversity may also associate with features linked to other microbiomes of the built environment, such as how dust microbiota are influenced by indoor chemistry and building design (e.g., antimicrobials used in cleaning products, presence of windows) [3, 4]. Aside from the limited understanding that antimicrobials incorporated into oral care products (e.g., chlorhexidine gluconate in mouthwash, nanoparticles on toothbrushes) influence bacterial counts on toothbrushes [18, 19], factors that more broadly impact the toothbrush microbiome are not well understood.

Here, we characterized the microbial diversity of 34 toothbrushes collected from randomly matched volunteers. Metagenomes of each sample were compared to those from the Human Microbiome Project (HMP-II) [20] and relevant indoor microbiome studies (e.g., microbiota associated with dust, sink tap water, and shower heads) [4, 21, 22] to uncover attributions to microbiota assembly and characterize potential emergence of antimicrobial resistance genes (ARGs) on toothbrushes. By evaluating metadata on dental hygiene and other personal information (e.g., diet, health, demographics) provided by participants, we tested the hypothesis that a combination of abiotic and biotic factors is important for shaping microbial communities and resistomes on toothbrushes.

## Results

### Members and sources of the toothbrush microbiota

Shotgun metagenomic sequencing of the DNA on toothbrushes yielded (5.07 ± 0.55) × 10^6^ quality reads per sample, providing estimated sequencing coverage of 83.1 ± 3.7% (*n* = 34; mean ± st. error) (Additional file 1: Fig. S1). There were 258 microbial species and 113 genera identified using an assembly-free marker-gene analysis of the microbial communities (i.e., MetaPhlAn2; Additional file 2: Table S1). Prominent phyla included *Actinobacteria*, *Firmicutes*, and *Proteobacteria*. The most abundant taxa were consistent with those identified by metagenome assembly, which yielded 110 bins assigned to 35 genera (Additional file 3: Table S2). At the genus-level, there was a strong correlation between the average relative abundance based on the marker-gene approach and the frequency among bins generated from metagenome assembly (*rho* = 0.706, *p* < 0.001; Additional file 4: Fig. S2).

**Fig. 1:**
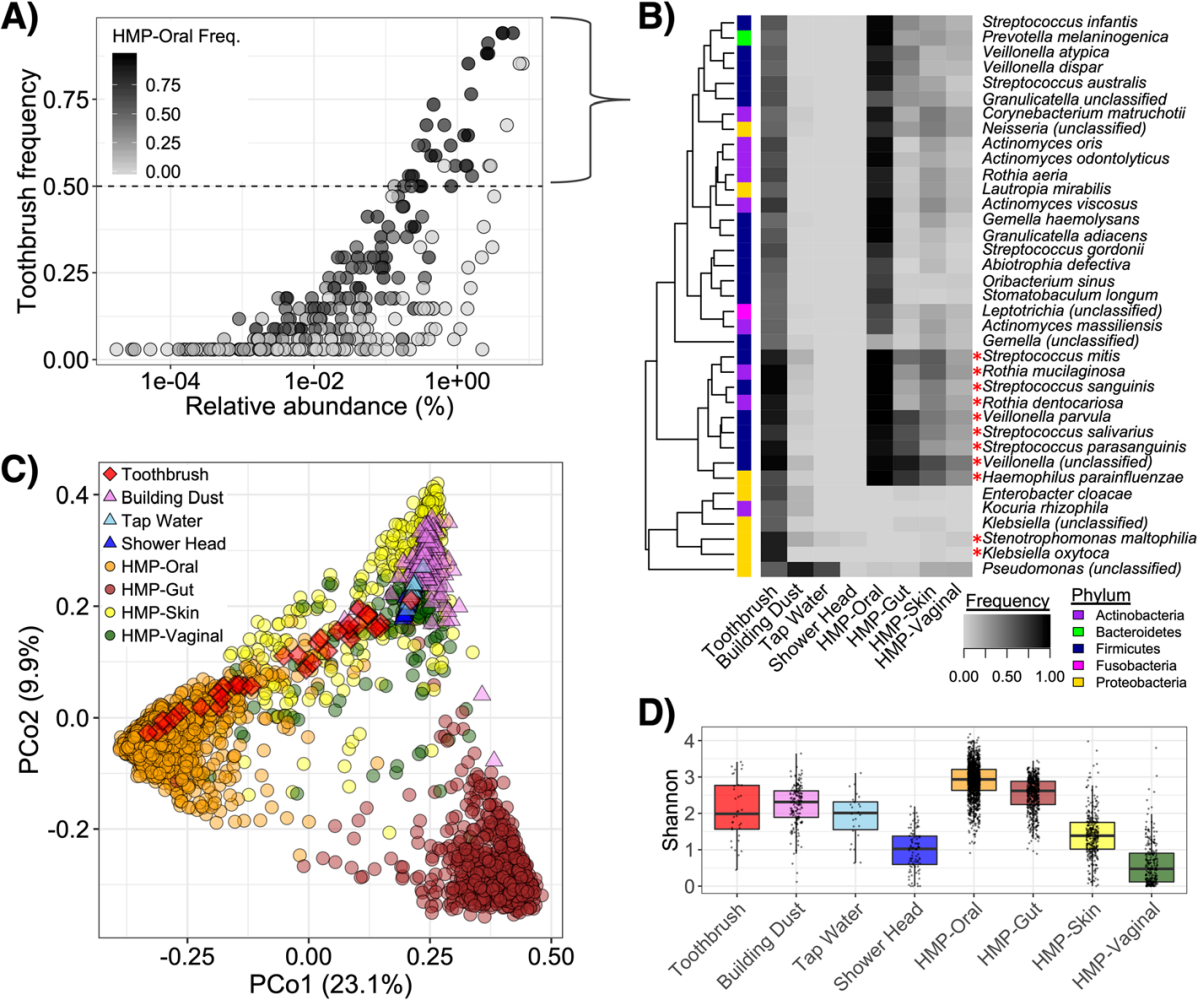
Toothbrush microbiota taxonomic diversity reflects a subset of the human oral microbiota with minor influence from other body sites and the built environment. **a** Frequency-abundance of toothbrush microbiota; color intensity corresponds to taxon frequency of detection in HMP-II [20] oral microbiome samples. **b** Frequency of detection of conserved toothbrush-associated taxa (i.e., those in at least 50% toothbrush samples) across different sample types, including toothbrushes, indoor dust [3, 4], tap water [21, 22], shower head biofilms [unpublished], and various body sites from the human microbiome project (HMP-II) [20]. Heatmap intensity reflects frequency of taxon detection in the respective sample types. Sidebar color corresponds to taxon phylum and an asterisk indicates member of the “core toothbrush microbiota” (i.e., in at least 75% samples). **c** PCoA displaying species-level beta-diversity across microbiota of toothbrushes, relevant environmental samples, and human microbiomes. Color/shape correspond to sample type. **d** Alpha-diversity (Shannon index) for each sample type

Toothbrush microbiomes contained a mix of human-associated taxa, primarily from oral body sites (Fig. 1a). The core microbiota (i.e., in > 75% of samples) included 8 predominant species frequently present in oral microbiomes (i.e., members of *Streptococcus*, *Rothia*, and *Veillonella*) and 2 others likely more associated with environmental origins (i.e., *Klebsiella oxytoca* and *Stenotrophomonas maltophila*) (Fig. 1b). More broadly, there were 37 relatively conserved bacterial species (i.e., in at least 50% of samples), of which 81.1% are common within the oral microbiome. These additional human-associated microbial species, many of which are found across multiple body sites despite occurring most frequently in oral microbiomes, included members of *Actinomyces*, *Corynebacterium*, and *Prevotella*, among others (Fig. 1b). The less-conserved toothbrush microbiota (i.e., in < 50% of samples) varied more in terms of possible origins, with only about half being frequently associated with the oral microbiome (Fig. 1a).

Despite notable overlap in membership with the human microbiota, toothbrush microbial communities had a widely distributed diversity (Fig. 1c). Variation in community composition and structure among toothbrush samples (Jaccard distance *=* 0.708 ± 0.029) exceeded that of person-to-person oral microbial communities (Jaccard distance *=* 0.563 ± 0.003) (*p* < 0.001; mean ± st. error). There was significant dissimilarity between the toothbrush microbiota and human oral (PERMANOVA *R*^2^ = 0.027), vaginal (PERMANOVA *R*^2^ = 0.062), skin (PERMANOVA *R*^2^ = 0.063), and gut (PERMANOVA *R*^2^ = 0.097) microbiota (*p* < 0.001 for all comparisons). Thus, oral microbiota were the least dissimilar taxonomic diversity with toothbrush microbial communities. Notably, out of the three primary oral sites sampled in the HMP-II (i.e., the buccal mucosa, supragingival plaque, and tongue), buccal mucosa was least dissimilar to toothbrushes (PERMANOVA *R*^2^ = 0.067, 0.131, 0.125, respectively; all *p* < 0.001), and they clustered closest together (Additional file 5: Fig. S3). Moreover, alpha-diversity (Shannon Index) of the toothbrush microbiota differed with that of each of the broadly characterized human body sites (Tukey’s *p* < 0.001 for all comparisons). While alpha-diversity on toothbrushes was greater than that of skin and vaginal microbiotas, it was lower than that of oral and gut microbiotas (Fig. 1d).

While about half of the toothbrush microbial communities clustered among oral microbiota in an ordination, another set appeared more closely related to skin microbiota and other microenvironments (Fig. 1c). The alpha-diversity of toothbrush-associated microbial communities was similar to that of indoor dust (Tukey’s *p* = 0.829) and tap water (Tukey’s *p* = 0.921), though not shower head biofilms (Tukey’s *p* < 0.001) (Fig. 1d). Nevertheless, the toothbrush microbiota had a structure and composition that was significantly different from dust (PERMANOVA *R*^*2*^ = 0.126, *p* < 0.001) and water (PERMANOVA *R*^2^ = 0.251, *p* < 0.001), as well as shower head biofilms (PERMANOVA *R*^2^ = 0.175, *p* < 0.001) (Fig. 1c, d).

**Fig. 2:**
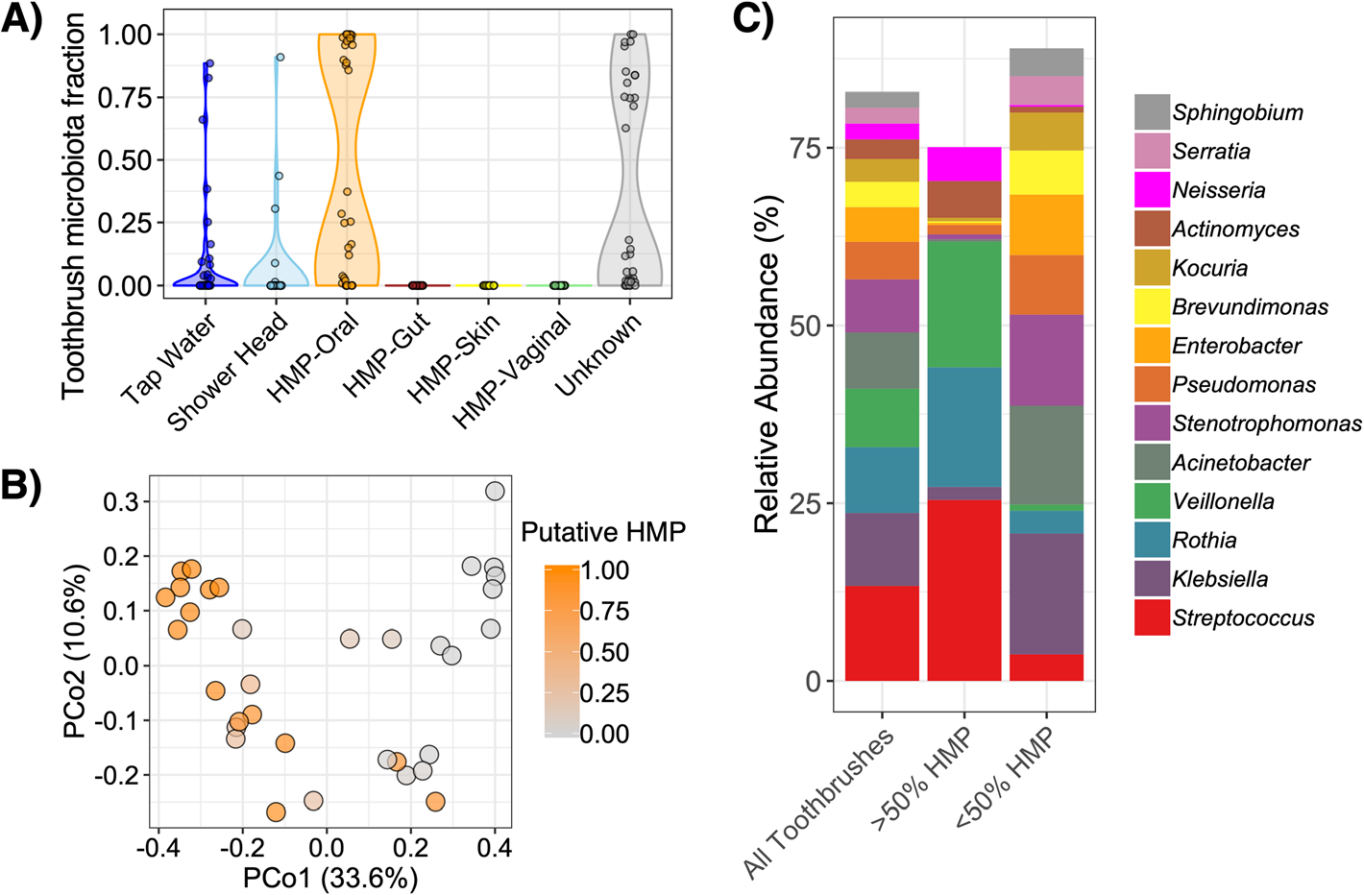
Toothbrush-associated microbiota are derived from a mix of prominent human and environmental taxa. **a** Distributions of putative origins of the toothbrush microbiota (genus-level) predicted by SourceTracker [23]. **b** PCoA displaying genus-level beta-diversity across toothbrush microbiota; color intensity indicates the cumulative fraction of taxa putatively derived from the human microbiome per sample. **c** Average relative abundances of highly abundant genera (i.e., > 2% average relative abundance) in all toothbrush metagenomes and those with putatively less than or greater than 50% microbiota derived from the human microbiome (i.e., primarily human-derived vs. non-niche-specific, respectively)

Both toothbrushes and indoor dust are microbial “sinks” containing a mixture of human-associated and environmental microbiota. SourceTracker analysis [23] suggested that, on average, 47.2% of the toothbrush microbiota was derived from humans (almost all oral origin), which is greater than the ~ 30% previously estimated for indoor dust [3, 4]. SourceTracker corroborated the PCoA ordination clustering revealing a bimodal distribution of putative human-derived taxa on toothbrushes, indicating that they were generally either covered with or lacking (i.e., > 75% or < 25%) such members (Fig. 2a). Accordingly, there was a strong relationship between taxonomic diversity on toothbrushes and the proportion of taxa attributed to the human microbiome, primarily the mouth (Fig. 2b; PERMANOVA *R*^*2*^ = 0.215, *p* < 0.001). The set of toothbrushes with greater than 50% of microbiota putatively derived from the human microbiome contained high relative abundances of members of *Streptococcus*, *Rothia*, *Veillonella*, *Actinomyces*, and *Neisseria* (Fig. 2c). Alternatively, key taxa from the set of toothbrushes containing less niche-specific microbiota included members of *Klebsiella*, *Acinetobacter*, *Stenetrophomonas*, *Pseudomonas*, and *Enterobacter* (Fig. 2c). The putatively non-human-derived fraction of the microbiota could be attributed to sink tap water (10.6%), shower head biofilms (5.2%), and possibly local environmental sources (i.e., 37.1% were of unknown origin), which may collectively drive the aforementioned large variation in taxonomic diversity (i.e., Fig. 1c). We note that since we used publicly available “training data” from different subjects and locations than the toothbrush samples, the SourceTracker predictions are approximate.

Overall, while toothbrushes contained a distinct microbiota (PERMANOVA *p* < 0.001) with most frequently occurring members putatively derived from the oral cavity (i.e., human oral microbiota was the greatest predicted source fraction and had the lowest PERMANOVA *R*^2^ compared to all other sample types), their alpha-diversity was most similar to other microbial communities found in the built environment.

### Resistome of the toothbrush microbiota

**Fig. 3:**
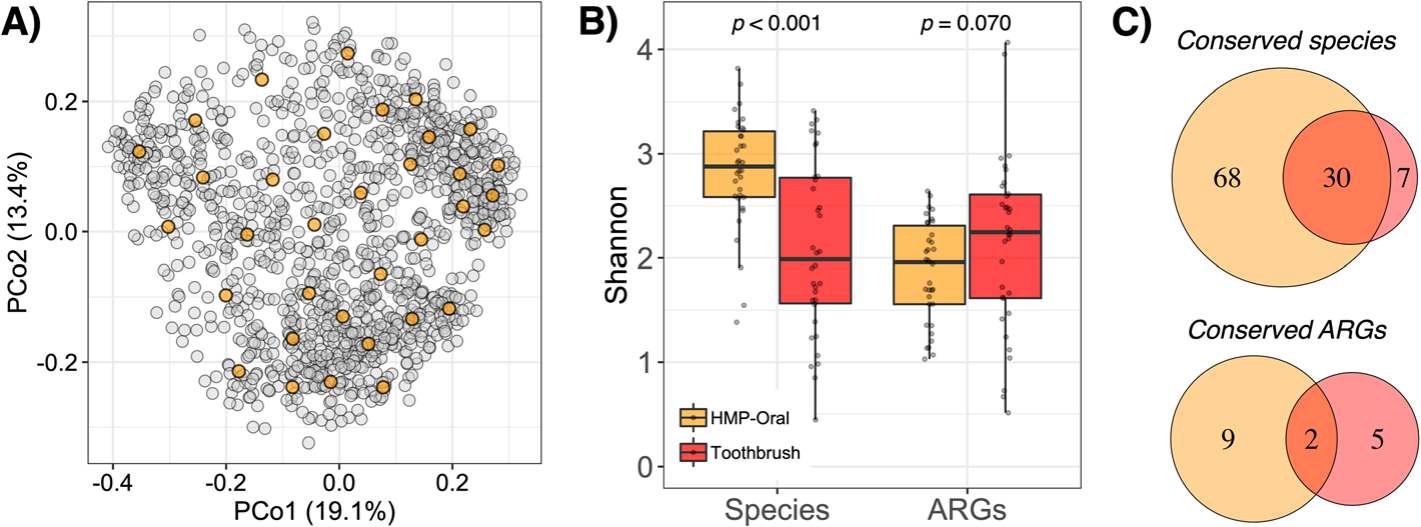
Although toothbrushes contained a less diverse microbiota and fewer commonly occurring antibiotic resistance genes (ARGs) than the oral microbiome, they had a more diverse resistome. **a** K-means clustering of all HMP-II oral sample taxonomic profiles (*n* = 1259; gray points) was used to generate centroids equal in size to the number of toothbrush samples (*n* = 34). Samples closest to the centroids (orange points) were selected for resistome analysis. **b** Shannon indices for overall taxonomic and ARG profiles for the subset oral samples (orange) and on toothbrushes (red). Mann-Whitney test *p* values for differences by sample type are displayed. **c** Overlap of conserved (i.e., detected in at least 50% respective samples) microbial species and antibiotic ARGs

The toothbrush metagenomes and the subset of oral metagenomes (Fig. 3a) contained 176 antibiotic resistance gene (ARG) protein families (Additional file 6: Table S3). The latter included samples from human buccal mucosa (*n* = 11), keratinized gingiva (*n* = 1), saliva (*n* = 1), supragingival plaque (*n* = 9), and tongue (*n* = 12) oral sites (Additional file 7: Table S4). ARGs varied in drug class and mechanism (e.g., antibiotic inactivation, efflux, target alteration) for predicted resistance (Table S3). We note that this list includes some widely conserved genes that confer intrinsic resistance and those that confer resistance when certain point mutations are present (e.g., *rpsJ*, *gyrB*); ARGs presented in our analysis refer only to a particular gene detected from the Comprehensive Antimicrobial Resistance Database (i.e., amino acid sequence predicted to confer resistance) [24]. Toothbrushes contained 158 ARG families with 21.8 ± 3.0 different families per sample, which was significantly greater than the 53 ARG families and 13.6 ± 1.1 different families per sample in oral metagenomes (mean ± st. error; *p* = 0.042). Unlike the microbiota taxonomic profiles, the ARG profiles of toothbrushes tended to be more diverse than those of oral-associated counterparts (Fig. 3b). Such disparity and the lack of correlation between toothbrush taxonomic and ARG alpha-diversity (*rho* = − 0.062; *p* = 0.720) suggests possible microbial selection based on resistome.

**Fig. 4:**
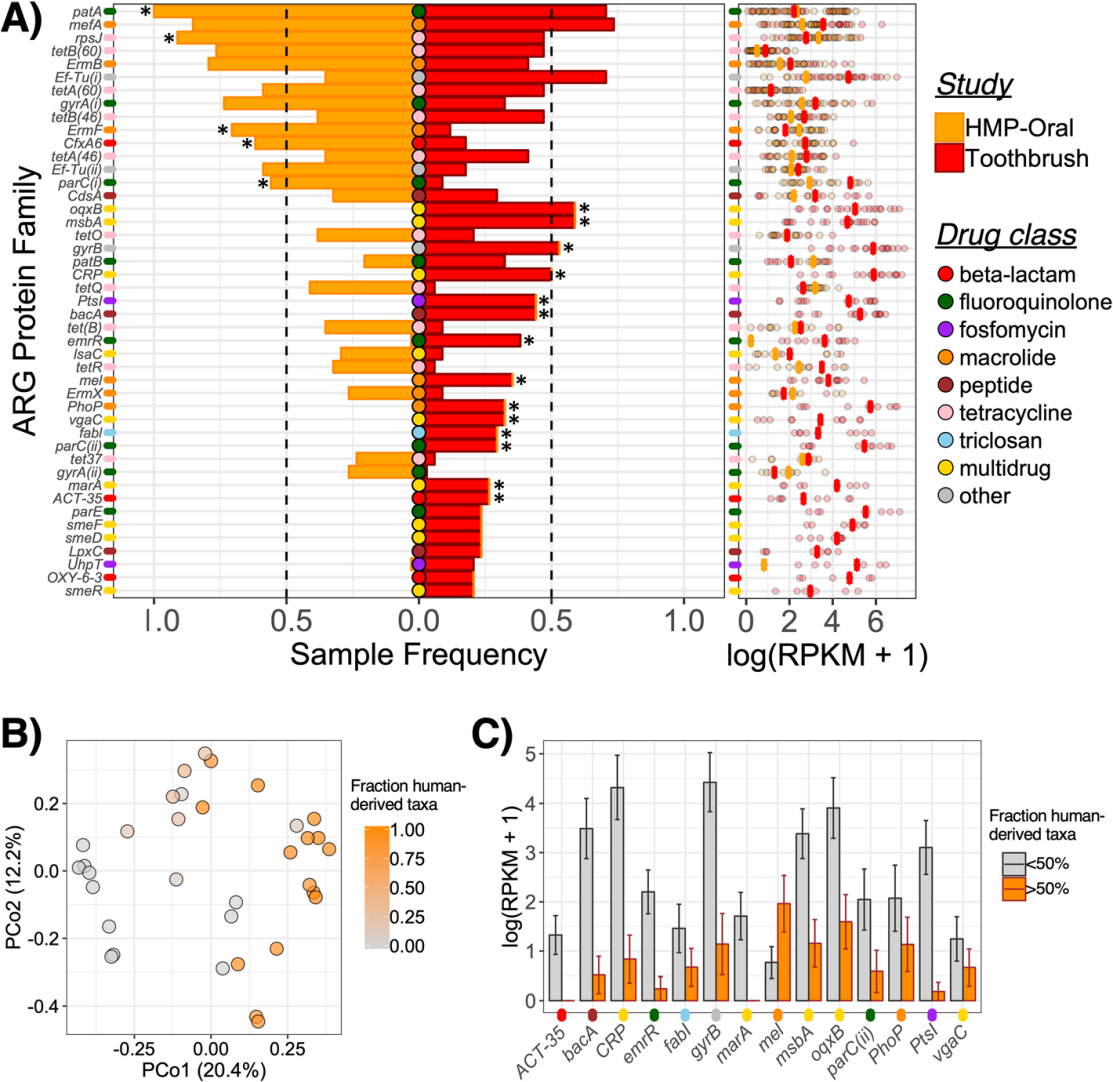
Enrichments in the toothbrush resistome correlate with the environmental-derived fraction of toothbrush microbial communities. **a** Left: Frequency of ARGs detected in oral (orange) and toothbrush (red) samples for ARGs present in > 10% of all samples (*n* = 68). Vertical dotted lines distinguish conserved ARGs (i.e., detected in at least 50% respective samples). An asterisk indicates enrichment by sample type (Bonferroni *q* < 0.05). Right: Log-transformed normalized RPKM counts for marker hits to each ARG protein family. Crossbar indicates median value for samples with > 0 counts. Axis tick colors correspond to ARG drug class. **b** PCoA displaying beta-diversity of toothbrush microbiomes based on normalized RPKMs of ARGs; color intensity indicates the cumulative fraction of taxa putatively derived from the human microbiome per sample (see Fig. 2a). **c** Log-transformed normalized RPKMs (average ± standard error) of ARGs that were enriched on toothbrushes (indicated in **a**) for toothbrush metagenomes containing less than 50% (gray) or greater than 50% (orange) microbiota putatively derived from the human microbiome

Toothbrush resistomes were enriched with more ARG protein families than oral resistomes (Fig. 4a) despite having fewer that were conserved (i.e., in at least 50% of samples) (Fig. 3c) and exhibiting more person-to-person variation (toothbrush Jaccard distance = 0.794 ± 0.034, oral Jaccard distance = 0.607 ± 0.032, *p* < 0.001). While oral samples were enriched with an ARG encoding resistance to tetracycline (*rpsJ*), toothbrushes were enriched with a variety of multidrug resistance genes (*oqxB*, *msbA*, *CRP*, *PhoP*, *marA*, *vgaC*) and those that confer resistance to triclosan (*fabI*) and fosfomycin (*PtsI*), among other mechanisms (*bacA*, *gyrB*). Both sample types further contained enrichments in different proteins that encoded similar resistances, i.e., to drug classes including fluoroquinolones (*patA* and a *parC* in oral; *emrR* and a *parC* in toothbrush), macrolides (*ermF* in oral; *mel* and *ErmX* in toothbrush), and beta-lactams (*CfxA6* in oral; *ACT-35* in toothbrush). For all conserved ARGs, log-transformed reads per kilobase per million (RPKMs) did not significantly differ by sample type (*q >* 0.05 for all) (Fig. 4a). Thus, compared to oral microbiomes, those on toothbrushes contained enrichments in the presence, but not copy number, of a variety of ARGs, some of which encoded resistances to drug classes not commonly conferred by oral microbiota. Overall, while both oral and toothbrush metagenomes contained a variety of ARGs, the latter were more diverse and uniquely associated with multidrug resistance and resistance to non-clinical antimicrobials (e.g., triclosan).

**Fig. 5:**
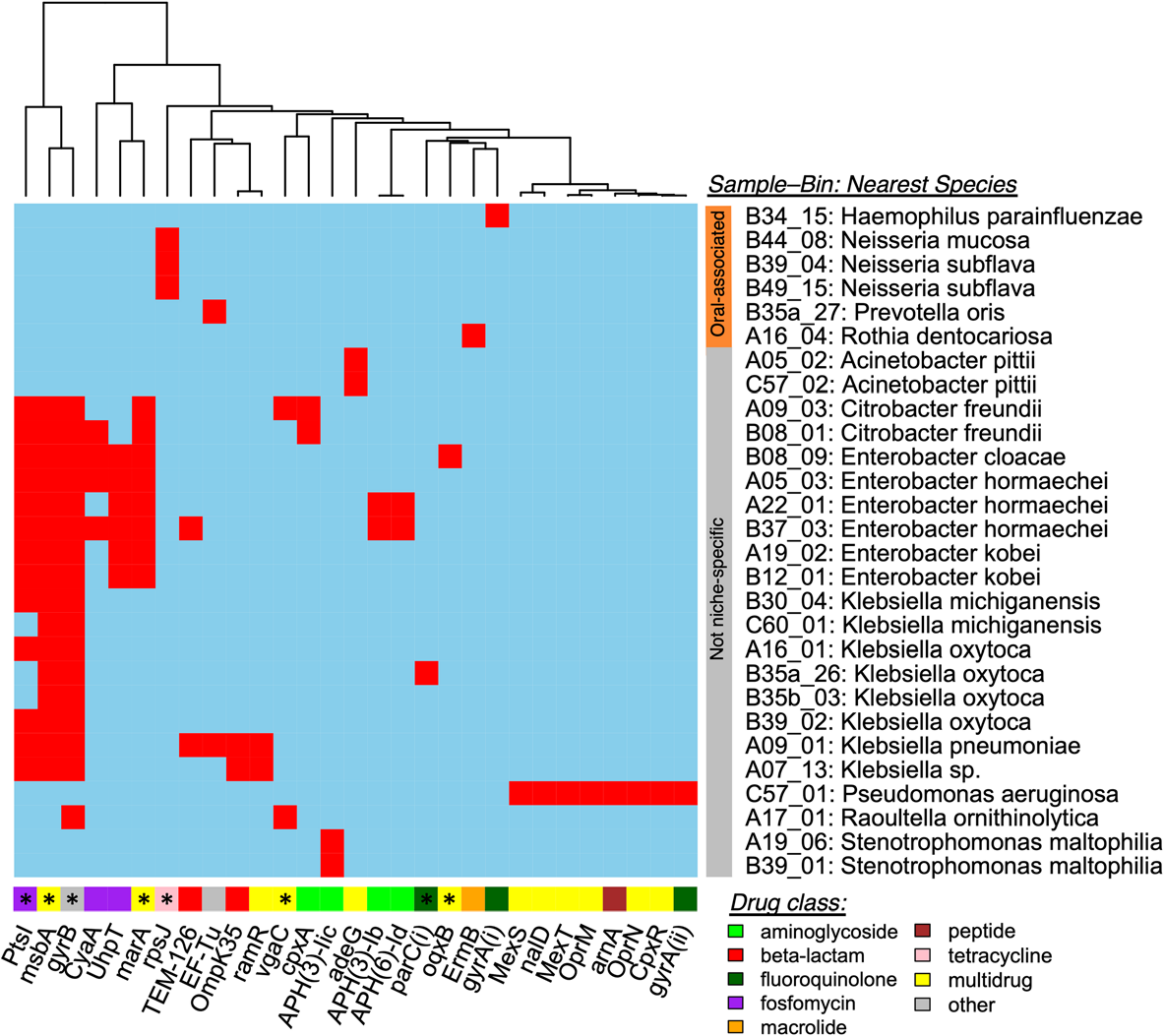
Metagenome-assembled genomes containing ARGs were most often linked to environment-associated taxa. Columns correspond to ARGs detected among bins (*n* = 29 ARGs); bottom side color corresponds to drug class, and asterisk indicates that the ARG had been enriched by toothbrush or oral-associated metagenome sample type (Bonferroni *q* < 0.05). Rows correspond to bins with at least one ARG detected (i.e., 28/110 bins); toothbrush sample ID, bin number, and the predicted bacterial species are displayed. Oral-associated or not niche-specific categories were assigned based on whether the bacterial species (or genus) was more or less abundant on toothbrushes that contained primarily oral-derived microbiota, as predicted by SourceTracker [23]

Enrichments within the toothbrush resistome appeared to be most attributable to versatile environmental-derived taxa. The fractions of toothbrush microbiota that were putatively sourced from the human microbiome vs. alternative origin significantly correlated with ARG profile beta-diversity (Fig. 4b; PERMANOVA *R*^2^ = 0.141, *p* < 0.001). Most of the enriched ARGs, except *mel*, were more abundant on toothbrushes that contained a microbiota primarily derived from such alternative sources (Fig. 4c). In agreement, metagenome assembly uncovered 28 bins containing 29 ARGs, most of which were assigned to environmental taxa with broad niche ranges, such as members of *Enterobacter*, *Klebsiella*, and *Pseudomonas* (Fig. 5). While several bins that were assigned to oral-associated taxa carried ARGs, notably intrinsic resistance (e.g., *Neisseria* encoding *rpsJ*), only those with unpredictable origins were found to encode possible multidrug resistance. Taken together, compared to the more conserved oral-associated taxa on toothbrushes, emergence of non-niche-specific strains within the microbial assemblages likely accounted for the unique toothbrush resistome.

### Factors shaping the toothbrush microbiome

**Fig. 6:**
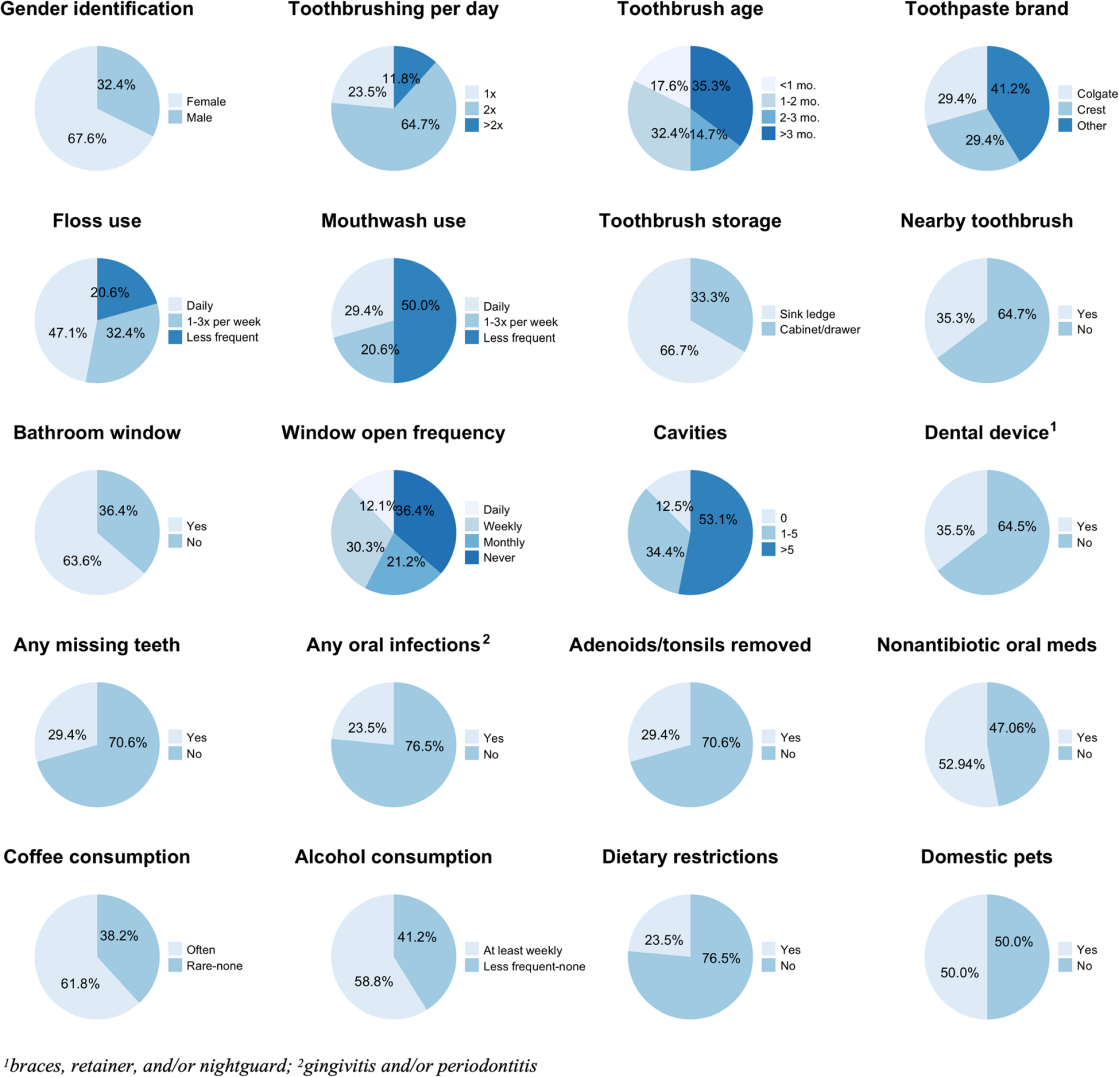
Summary statistics for subject population (*n* = 34). Participant information regarding oral hygiene, health and activity, and bathroom attributes. These metadata were selected from all survey responses (*n* = 20/40) for statistical analysis with toothbrush microbiome data, as they had relatively well-balanced responses (i.e., at least 20% for more than one categorical answer in the respective question)

We inferred a variety of factors that may influence the toothbrush microbiome structure and composition from the participant metadata (Fig. 6). Beta-diversity across microbiota was significantly, yet weakly associated with gender identity (PERMANOVA *R*^2^ = 0.055, *p* = 0.031), missing teeth (PERMANOVA *R*^2^ = 0.078, *p* = 0.004), and adenoid/tonsil removal (PERMANOVA *R*^2^ = 0.072, *p* = 0.010) (Additional file 8: Fig. S4). Similarly, beta-diversity across ARG profiles was weakly linked to having missing teeth (PERMANOVA *R*^2^ = 0.053, *p* = 0.030) and adenoid/tonsil removal (PERMANOVA *R*^2^ = 0.058, *p* = 0.013) (Additional file 8: Fig. S4). Perhaps related, the putative human-derived content of toothbrush microbiota was inversely proportional to having adenoids/tonsils removed (*p* = 0.011). No other metadata from the exploratory analysis appeared to be significantly linked to predicted source content of the toothbrush microbiota, perhaps a reflection of the relatively small sample size and wide variation across toothbrush microbiomes.

**Fig. 7:**
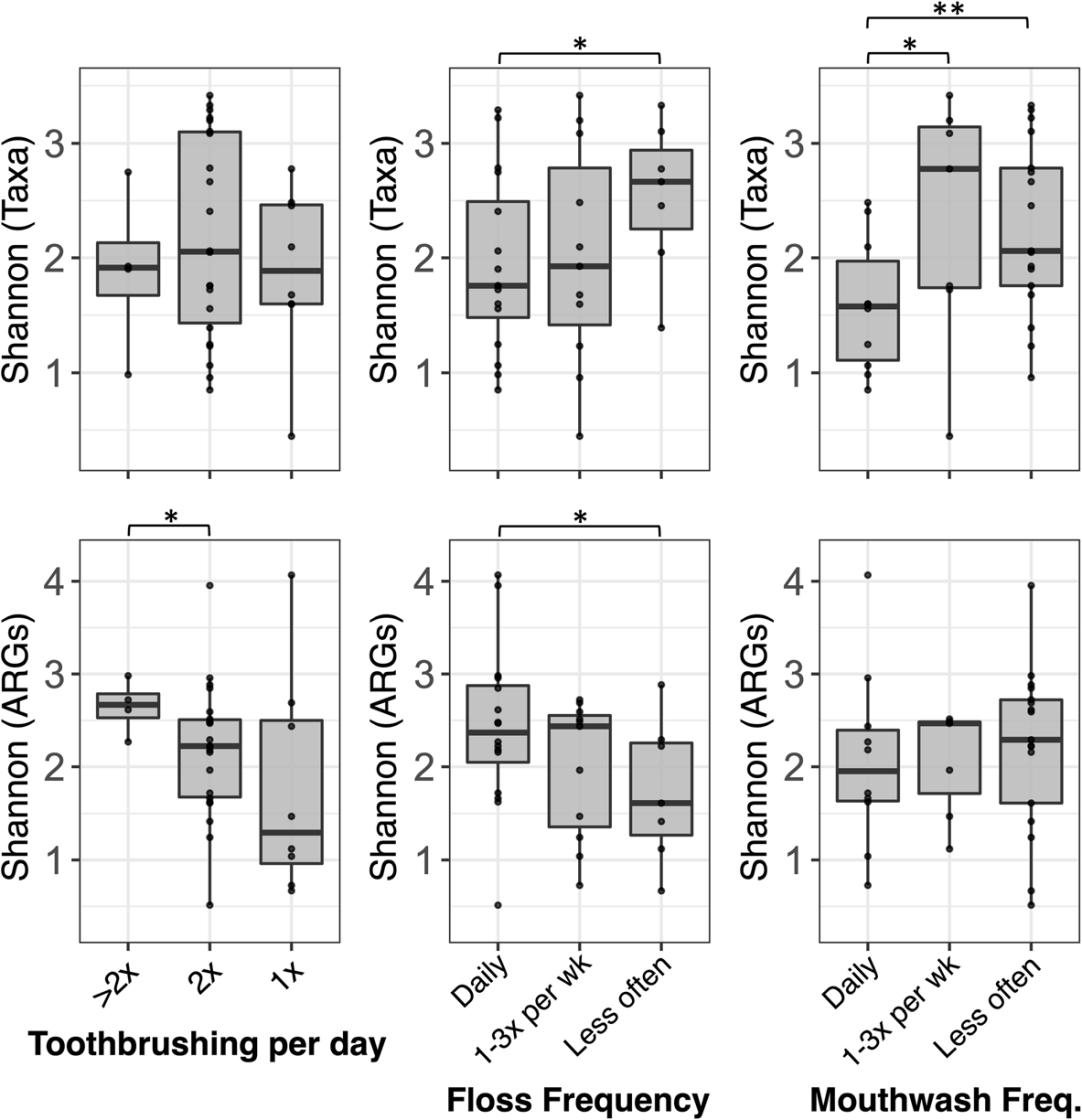
Trends for possible dental hygiene associations with diversity of toothbrush microbiome taxonomic (top row) and ARG (bottom row) profiles. Comparisons yielding Mann-Whitney test *p ≤* 0.05 or *p ≤* 0.1 are displayed as “**” or “*,” respectively

More broadly, alpha-diversity of microbial taxonomic and ARG profiles appeared to be weakly related to oral hygiene (Fig. 7). While there were trends for frequencies of flossing and mouthwash use being inversely associated with taxonomic alpha-diversity, possibly due to removal of potential microbial colonists, an increased frequency of toothbrushing and flossing positively associated with resistome alpha-diversity (Fig. 7). In addition to dental hygiene, microbiota alpha-diversity significantly correlated with gender identity (*p* = 0.045). Toothbrushes belonging to people that identified as women contained more diverse microbial communities than those belonging to men (Additional file 9: Fig. S5), even though gender identity was not significantly correlated with taxonomic (*p* = 0.144) or ARG profile (*p* = 0.418) alpha-diversity of oral metagenomes. Moreover, resistomes on toothbrushes belonging to women and persons taking non-antimicrobial oral medications, those that were stored on sink-top ledges, and those in bathrooms without windows, or with windows that were more frequently closed, exhibited trends for slightly more diverse ARG profiles than respective counterparts (Additional file 10: Fig. S6).

**Fig. 8:**
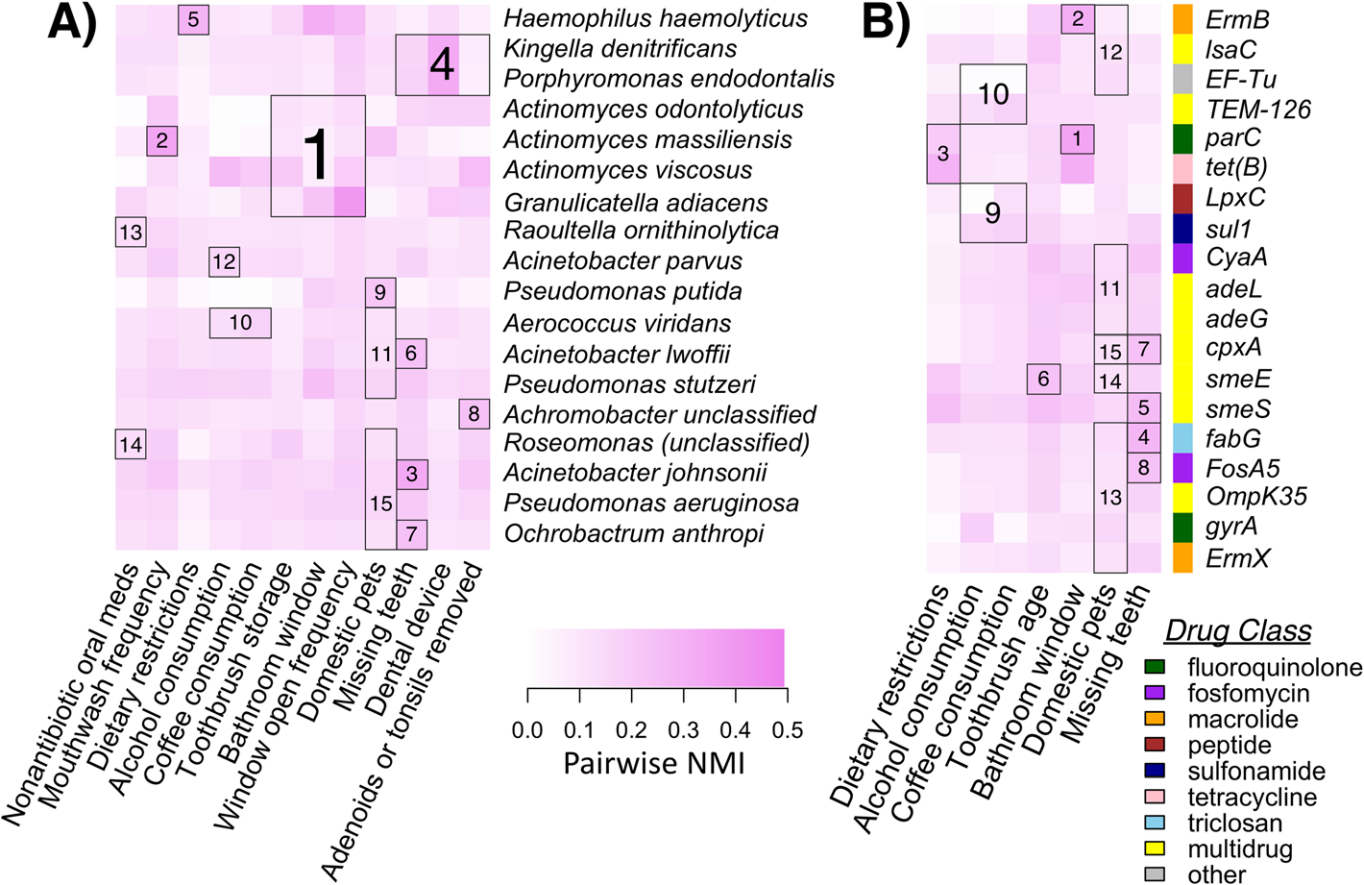
Significant associations between metadata and relative abundances of bacterial species (**a**) or RPKMs of ARGs (**b**) in toothbrush microbiomes based on Hierarchical All-against-All association (HAllA) testing. All metadata presented in Fig. 4 (*n* = 20 variables) were included in the HAllA analyses and those with significant association (*q* < 0.1) to at least one taxon/ARG are presented in the respective figure panels (i.e., 12/20 and 7/20 variables associated with particular taxa and ARGs, respectively). Significant clusters of related features (*q* < 0.1) are outlined and ranked based on hierarchy of similarity scores. Grid color intensity corresponds to normalized mutual information similarity metric. Sidebar colors in **b** correspond to the ARG drug class

Regarding specific microbial responses to putative selective pressures, hierarchical all-against-all significance testing identified 12 variables significantly associated with relative abundances of 18 different species (Fig. 8a). For example, bathroom attributes (i.e., window presence, window open frequency, toothbrush storage location) correlated with relative abundances of commonly occurring bacterial species within *Actinomyces* and with *Granulicatella adiacens*. Whether or not a person had domestic pets in their home, had any missing teeth, or had adenoids or tonsils removed correlated with members of *Acinetobacter*, *Kingella*, *Porphyromonas*, and *Pseudomonas*, among other taxa. We further uncovered associations between relative abundances of 19 different ARG protein families and 7 variables, again including bathroom attributes, missing teeth, and domestic pets (Fig. 8b). While most of the ARGs that correlated with features associated with multidrug resistance, other drug classes included fluoroquinolones, fosfomycin, macrolides, tetracyclines, triclosan, and sulfonamides (Fig. 8b). Thus, a complex combination of personal health, dental hygiene, and environmental variables likely shapes taxonomic diversity and antimicrobial resistance in toothbrush microbiomes.

## Discussion

Toothbrushes are an interface for microbiota derived from human hosts and the surrounding environment. Most of the prominent members of the toothbrush microbiome were those that are common in oral microbiomes [20]. Several conserved oral-associated taxa, such as *Actimomyces*, *Corynebacterium, Haemophilus*, *Neisseria*, and *Streptococcus*, play important roles in biofilm attachment and development of supragingival plaque [25]. Mechanisms underlying biofilm formation may have implications for colonization of toothbrush microenvironments. Although there was broad taxonomic overlap with the oral microbiome, especially with buccal mucosa sites, toothbrushes harbored more heterogenous and less diverse microbial communities. Trends for wide variation across toothbrush microbiomes were similar to what has been described on dust or surfaces in the built environment, i.e., other “sinks” for human-derived microorganisms [3, 4, 7]. Indeed, many of the less niche-specific taxa observed on toothbrushes are found throughout indoor spaces, e.g., members of *Enterobacter*, *Kocuria*, *Pseudomonas*, and *Stenotrophomonas* [5, 26–29]. Collectively, toothbrush microbial communities ranged from robust subsets of oral microbiota to mixed assemblages containing more abundant case-specific or environmental strains.

Despite lower taxonomic alpha-diversity, toothbrush metagenomes contained more antimicrobial resistance genes (ARGs) than oral counterparts. While the majority of the conserved microbial taxa on toothbrushes were common members of the oral microbiota, communities that were primarily composed of putatively non-human-derived taxa encoded, on average, higher counts of toothbrush-enriched ARGs. Metagenome assembly validated that most of the toothbrush-associated taxa carrying such ARGs, among others, associated with microbial communities dominated by non-niche-specific microorganisms. Potential ARG-carrying colonists may have been incorporated from the bathroom sink and local surroundings. For example, toothbrush-associated *oqxB*, which may confer multidrug resistance (e.g., nitrofuran, tertracycline, quinolone), is common in tap water resistomes [21]. Stressors in the built environment (e.g., temperature variations, exposures to anthropogenic chemicals, periodic desiccation) may play important roles in elevating toothbrush resistomes, at least for key members that emerge within the microbial community from the environment. For example, triclosan and other anthropogenic chemicals in personal care products (e.g., soaps, shampoo, lotions) that have become widespread in indoor spaces have been linked to multidrug resistance in dust-associated microbial communities [3, 4]. Toothbrushes may harbor some similar microbial colonists, especially considering ARG enrichments reflected a variety of multidrug efflux genes along with putative conferred resistance to triclosan, i.e., *fabI* and *fabG* [30]. In fact, several oral care products even contain anthropogenic antimicrobials (e.g., triclosan in toothpastes, nanoparticles on toothbrushes) that may accumulate on and/or leach from bristles [18, 31]. While such compounds are intended to lessen bacterial load to promote oral health, there could be undesirable consequences such as induced selection of opportunistic oral pathogens (e.g., as noted for *Prevotella* [12, 19]) or ARGs. Moreover, since most toothbrush-enriched ARGs were still present in microbial communities containing primarily human-derived taxa, albeit to a lesser extent than those composed of mostly case-specific or environmental strains, unique toothbrush resistomes may be partially related to selection of human-derived taxa best suited for survival under harsh built environment conditions.

Microbial communities on toothbrushes appeared to weakly associate with several factors previously shown to correlate with microbiomes of the oral cavity, e.g., gender identity, missing teeth, adenoid/tonsil removal, and ownership of domestic pets [32–36]. While dental hygiene frequency (i.e., toothbrushing, flossing, mouthwash use frequencies) was inversely related to microbial taxonomic diversity on toothbrushes, which was consistent with an earlier report of mouth-rinse lowering bacterial counts on toothbrushes [19], it was positively linked to the resistome. Although plausible that there could have been less accumulation of human-derived taxa on toothbrushes of people that more frequently practice comprehensive dental hygiene (e.g., less plaque on teeth leading to less transfer to toothbrushes), putative source fraction of the toothbrush microbiota did not significantly associate with oral care. Thus, the relationship between more frequent dental hygiene and a broader resistome suggests possible selection among the mixture of human- and environmental-derived microbiota. The positive association with the toothbrush resistome may also partially be due to more frequent incorporation of ARG-carrying members of oral microbiota or a raised surrounding dust microbial community. Links between toothbrush microbiomes and health, at least as a potential biomarker, warrant investigation with a larger cohort with balanced clinical metadata.

Correlations between toothbrush microbiomes and features of the surrounding environment (i.e., window presence and frequency of opening) are consistent with previous work showing building design to weakly influence indoor microbial communities [37]. Although there is a strong desire to for using “bioinformed design” to create healthier indoor microbiomes [38], the vast majority of observed indoor microbiota are likely dead or metabolically inactive [39], in no small part due to lack of moisture. However, there are niches in the built environment that, like toothbrushes, are frequently wetted (i.e., sponges, dishwashers, washing machines) [40–42]. The toothbrush microbiota is similar to these in that they appear to be “hotspots” for putative opportunistic pathogens and antimicrobial resistance, yet different in particular microbial membership (i.e., toothbrushes contain primarily oral-derived taxa) and in biofilm dynamics. Since our exploratory analysis was based on correlation and did not control for variables that may drive the microbiome, future studies are needed to better understand how we may be able to target design/operation/use of different components of the built environment to promote microbial diversity that limits pathogen and antimicrobial resistance on toothbrushes.

As the scope of our pilot study was to define the toothbrush microbiome and potential driving factors, limitations included the relatively small sample size (*n* = 34) and the lack of paired donor samples. Perhaps a larger cohort would improve effect sizes for the weak, yet potentially important trends uncovered in this pilot study (e.g., microbiome associations with oral hygiene practices, missing teeth, adenoid/tonsil removal, presence bathroom windows, toothbrush storage location). After all, although indoor microbial communities are impacted largely by the environment, such features often have small or negligible associations with community metrics [43]. Moreover, the comparisons made between toothbrush microbiomes and human-associated and built environment counterparts from separate studies are “high-level” due to site-specificity of microbial strains and possible confounding variables across datasets (e.g., variation in treatment of actual sink water in subject households vs. tap water in reference datasets, batch effects in sequencing preparation). For example, some of the putative oral-derived microbiota may have been overestimated and rather had come from other human body sites. While members of the toothbrush microbiota putatively derived from the human microbiome were most common across oral metagenomes, hence the human-fraction predictions made by SourceTracker [23], many of the same microbial species occur in skin, vaginal, and gut microbiomes as well (e.g., *Streptococcus* and *Veillonella*). Likewise, some of the prominent taxa on toothbrushes that contained mostly non-human-derived microbiota may still associate with human body sites, e.g., potential enteric strains of *Enterobacter* or *Klebsiella*. Future longitudinal studies monitoring microbiomes of toothbrushes and donors, perhaps incorporating genome-level sequence variant comparisons for tracking, would be essential for making stronger conclusions about community assembly on toothbrushes and potential driving factors. Nevertheless, considering distinctions from the human microbiota that may indicate local selection, toothbrush microbiomes appear to be an important reservoir for antimicrobial resistance. It remains to be determined whether this sink may, in turn, become a relevant source for ARG dissemination in the human microbiome and indoor environment.

## Conclusions

We applied a metagenomic approach to define the microbial communities and resistomes on toothbrushes that were collected from 34 individuals. By comparing these data with previous human- and built environment microbiome studies, we show that the microbial communities on toothbrushes are composed of dominant oral-derived taxa in combination with more versatile environmental strains. Although toothbrushes had a less diverse microbiota than oral microbiomes, they contained more diverse resistomes with key gene enrichments (e.g., multidrug efflux, triclosan resistance) that appeared to be mostly attributable to the non-niche-specific, perhaps environmental-derived, fraction of microbiota. Dental hygiene and bathroom features, among other factors, weakly associated with the toothbrush microbiomes. Overall, the taxonomic and ARG diversity within these microbial communities reflects a unique reservoir where human oral and environmental microbes encounter each other, which could potentially enable the transfer of ARGs.

## Methods

### Sample collection

Participants were recruited through Research Match (https://www.researchmatch.org) to donate their toothbrush for microbiome analysis and provide accompanying metadata regarding demographics, dental hygiene, diet, etc. Recruitment took place in three cycles, each a 1-month time window, for adults aged 18–65 years living up to a 100-mile radius from Northwestern University, Evanston, IL, USA. After completing the personal information survey online in REDCap (Additional file 11: Dat. S1), volunteers were mailed kits containing materials to send in their toothbrush via overnight shipping. All participants were identified via a blind cataloging system to maintain anonymity. In total, we received 34 toothbrush samples with corresponding metadata.

### Sample preparation and sequencing

Samples were stored at 4 °C after arrival for no longer than 24 h. Using aseptic technique, the head of each toothbrush was removed with shears, which had been treated with DNA AWAY™ (Molecular BioProducts™), and placed into a sterile 50-mL conical tube. Toothbrush heads were submerged in 15 mL PBS with 0.01% Tween 80, vortexed for 10 s, and placed on an orbital shaker at 25 °C and 180 revolutions per minute (RPM) for 10 min. Samples were sonicated with a Model 120 Sonic Dismembrator (ThermoFisher Scientific, Waltham, MA, USA) set to the following: duration of 2 min, pulse of 10 s on and 10 s off, amplitude of 40%, and power of 4 W and up to 450 J. Processed samples were stored at − 20 °C until further analysis.

Prior to DNA extraction, sample tubes were thawed at room temperature and 1.0 mL of supernatant was aliquoted and centrifuged at 10,000*g* for 3 min. The supernatant was decanted leaving a pellet and approximately 25 μL of solution. DNA was extracted using the MasterPure™ Complete DNA and RNA Purification Kit (Lucigen, Middleton, WI, USA). DNA quality was measured with a Synergy HTX Multi-Mode Reader (BioTek, Winooski, VT, USA) and was considered acceptable for downstream analysis if the 260/280 ratio was between 1.7 and 2.1. DNA concentrations were quantified using the Quant-iT™ dsDNA Assay Kit (ThermoFisher Scientific, Waltham, MA, USA) [44]. Seven of the 34 samples were originally found to have relatively low yields of DNA that were below requirements for the library prep (0.2 ng uL^−1^); these samples were re-processed with the same steps above using 5 mL of toothbrush head supernatant.

Metagenomic libraries were prepared using the Nextera XT and Index Kit v2 set A (Illumina, San Diego, CA, USA). Fragment analysis was performed using an Agilent 2100 Bioanalyzer (Santa Clara, CA, USA). Libraries were normalized, pooled, and sequenced for 2 × 150 bp reads on an Illumina HiSeq 4000 platform (San Diego, CA, USA). Sequencing was performed by the NUSeq Core facility.

### Metagenome data processing and analysis

Data were analyzed using the Genomics Compute Cluster on Quest. Paired-end reads were pre-processed and merged using KneadData v0.6.1 (http://huttenhower.sph.harvard.edu/kneaddata) with default parameters. Reads were filtered and trimmed, and low-quality reads, human sequences, and sequences present in negative controls from DNA extraction and library prep were removed.

Metagenome coverage for each sample was estimated with Nonpareil v3.303 [45]. Toothbrush microbial community taxonomic compositions and abundances were determined with MetaPhlAn2 v2.7.7 [46]. Putative source fractions of the microbiota at the genus-level were estimated with SourceTracker [23]. We trained the Bayesian model to test for potential sources from tap water [21, 22] and shower heads (unpublished) as well as human-associated oral, skin, vaginal, and gut microbiomes (HMP-II) [20]. Antimicrobial resistance gene (ARG) profiles of the toothbrush metagenomes were determined with ShortBRED v0.9.5 [47]; *shortbred_quantify* was run with *shortbred_identify* markers (marker length > 30 amino acids) constructed from the Comprehensive Antibiotic Resistance Database v3.0.2 [24] and UniRef90 as reference [48].

Toothbrush metagenomes were processed for assembly and binning using KBase [49]. Raw reads were imported as paired-end libraries and processed for quality control with Trimmomatic v0.36 [50]. Cleaned reads were assembled with MegaHit v1.2.9 [51] and contigs were binned with MaxBin2 v2.2.4 [52]. Quality of bins was determined using CheckM v1.0.18 [53], and those with minimum 75% completeness and maximum 10% contamination were exported to be further processed with CGE Kmer Finder 3.2 (https://cge.cbs.dtu.dk/services/KmerFinder/) for taxonomic identification. Frequencies of taxa identified among bins were compared with those identified using the aforementioned marker-gene approach (i.e., MetaPhlAn2).

Statistical analyses and data visualization were performed in R v3.6.0. Vegan v2.5-5 [54] was used to compute microbiome diversity metrics. Differences in microbiome alpha-diversity (Shannon index) based on sample type—toothbrush, human body site [20], tap water [21, 22], shower heads [unpublished], and indoor dust [3, 4]—were determined with an ANOVA and Tukey’s post-hoc test. Beta-diversity was evaluated with principal coordinate analysis (PCoA) and permutational multivariate ANOVA (PERMANOVA), using Jaccard’s index as the diversity metric. The same metric was used to determine the association between toothbrush microbial communities and putative source fraction derived from human microbiota.

The resistomes associated with toothbrushes were compared to those of human oral samples using an equal-sized subset from the HMP-II (i.e., *n* = 34 for each sample type). The subset of oral microbiome samples contained those closest in ordination distance to 34 centroids generated by k-means clustering taxonomic profiles of all HMP-II oral samples. Since the full sample set (*n* = 1259) contained metagenomes from a variety of oral sites (e.g., buccal mucosa, tongue, supragingival plaque, keratinized gingiva) that toothbrushes may come in contact with, all oral sample types were included in the clustering analysis. The metagenome sequence files associated with selected samples were downloaded from the sequence read archive (SRA) and processed with ShortBRED as described above. Spearman-rank correlation between alpha-diversity of taxonomic and ARG profiles of toothbrushes was determined. Additionally, overlap in the “core” resistome of toothbrushes and oral samples (i.e., detected in over 75% of the respective samples) was evaluated. For all ARGs detected, a generalized linear model (GLM) with binomial error distribution was used to determine differences in frequency of occurrence based on sample type. The resulting *p* values were adjusted to *q* values by Bonferroni correction, and associations with *q* < 0.05 were considered significant. Log-transformed reads per kilobase per million mapped reads (RPKM) by sample type were further compared with the Mann-Whitney test.

The association between beta-diversity of toothbrush resistomes and putative source fractions of the toothbrush microbiota was evaluated with principal coordinate analysis (PCoA) and permutational ANOVA (PERMANOVA), using Jaccard’s index as the diversity metric. Abundances of the toothbrush-associated ARGs (i.e., log-RPKMs) were compared across microbial communities containing greater than or less than 50% putatively human-derived microbiota using the Mann-Whitney test. Moreover, metagenome bins were processed with ShortBRED as described above to link identified ARGs to specific taxa.

All participant metadata were extracted from the REDCap online interface. While approximately 40 questions were asked in the survey (Additional file 11: Dat. S1), only those receiving relatively balanced responses (i.e., at least 20% for more than one categorical answer in the question) were included in the statistical analysis (Fig. 4). Correlations between these metadata and toothbrush microbiome alpha-diversity (Shannon Index) were evaluated with the Mann-Whitney test or Kruskal-Wallis test. Metadata associations with microbiota and resistome beta-diversity were assessed with PCoA and PERMANOVA, using Jaccard’s index as the diversity metric. Although alpha was set at 0.05 for significance, given this was a pilot study exploratory analysis with relatively small sample size, we noted additional trends up to the *p* < 0.1 threshold. Hierarchical all-against-all significance testing (HAllA v0.7.18; http://huttenhower.sph.harvard.edu/halla) was further used to determine potential associations between metadata and detected microbial community membership and ARGs. Clusters of related features with false discovery rate-adjusted *q* values < 0.1 were considered significant.

## Supplementary Information

**Additional file 1: Figure S1.** Nonpareil curves illustrating sequencing coverage of the toothbrush metagenomes. Solid and dotted lines correspond to observed and predicted redundancy, respectively.

**Additional file 2: Table S1.** Frequency and relative abundance of microbial species detected in toothbrush metagenomes. Conserved taxa (i.e., at least 50% of samples) are in bold font.

**Additional file 3: Table S2.** Metagenome-assembled genomes with > 75% completeness and < 10% contamination. Taxonomic assignments and summary statistics reflect the top match from CGE Kmer Finder 3.2 (https://cge.cbs.dtu.dk/services/KmerFinder/).

**Additional file 4: Figure S2.** Relationship between genera relative abundances within toothbrush microbial communities predicted using the marker-gene approach (i.e., MetaPhlAn2) and genera frequencies among metagenome-assembled genomes. Genera with over 5% from either category are listed.

**Additional file 5: Figure S3.** PCoA displaying species-level beta-diversity across microbial communities on toothbrushes and those from specific oral sites (HMP-II). Colors/shapes corresponds to sample type.

**Additional file 6: Table S3.** ARG protein families detected on toothbrushes and in the subset of HMP-II oral samples. Drug class and mechanism of resistance are in accordance with the Comprehensive Antibiotic Resistance Database [24]. Sample detection frequency, RPKM mean and standard error, and GLM p and q values are listed. ARGs enriched (q < 0.05) in toothbrush or oral samples are indicated in blue and green font, respectively.

**Additional file 7: Table S4.** Accession numbers and available metadata for the oral metagenomes used in the resistome analysis, which correspond to the subset in Figure 3a.

**Additional file 8: Figure S4.** Metadata that may associate with beta-diversity of toothbrush microbiota (left panels) and toothbrush resistomes (right panels), i.e., factors from the set of 20 variables displayed in Figure 4 that had a PERMANOVA p < 0.1.

**Additional file 9: Figure S5.** Metadata that may associate with alpha-diversity of toothbrush microbiota taxonomic profiles; i.e., factors from the set of 20 variables displayed in Figure 4 that had a Mann-Whitney or Kruskal-Wallis test p < 0.1.

**Additional file 10: Figure S6.** Metadata that may associate with alpha-diversity of toothbrush resistomes, i.e., factors from the set of 20 variables displayed in Figure 4 that had a Mann-Whitney or Kruskal-Wallis test p < 0.1.

**Additional file 11: Dat. S1.** Sample survey questionnaire that was used to collect metadata.

## Data Availability

Toothbrush metagenome sequence data generated and analyzed are available in the NCBI Sequence Read Archive under Bioproject number PRJNA596937. HMP-II metagenome sequence data used in the resistome analysis are available in the NCBI SRA under accession numbers indicated in Additional file 7: Table S4. All bioinformatics scripts and generated data that may be used to reproduce our analyses are available at https://github.com/hartmann-lab/Toothbrush_Microbiome_Project.

## Abbreviations

ANOVA: Analysis of variance
ARGs: Antimicrobial resistance genes
GLM: Generalized linear model
HAllA: Hierarchical All-against-All association test
HMP: Human Microbiome Project
PBS: Phosphate buffer solution
PCoA: Principle coordinate’s analysis
PERMANOVA: Permutational multivariate ANOVA
RPKM: Reads per kilobase per million
RPM: Revolutions per minute
SRA: Sequence read archive

## References

[1] Ben Maamar S, Hu J, Hartmann EM. Implications of indoor microbial ecology and evolution on antibiotic resistance. J Expo Sci Environ Epidemiol. 2019. 10.1038/s41370-019-0171-0. 10.1038/s41370-019-0171-0 PMC8075925 31591493

[2] Gilbert JA, Stephens B (2018). Microbiology of the built environment. Nat Rev Microbiol..

[3] Hartmann EM, Hickey R, Hsu T, Betancourt Román CM, Chen J, Schwager R (2016). Antimicrobial chemicals are associated with elevated antibiotic resistance genes in the indoor dust microbiome. Environ Sci Technol..

[4] Fahimipour A, Ben Mamaar S, McFarland A, Blaustein RA, Chen J, Glawe A, et al. Widespread antimicrobial chemicals influence the structure and function of indoor microbial communities. mSystems. 2018; (accepted). 10.1128/mSystems.00200-18 PMC6290264 30574558

[5] Lax S, Sangwan N, Smith D, Larsen P, Handley KM, Richardson M (2017). Bacterial colonization and succession in a newly opened hospital. Sci Transl Med..

[6] Blaustein RA, McFarland AG, Ben Maamar S, Lopez A, Castro-Wallace S, Hartmann EM (2019). Pangenomic approach to understanding microbial adaptations within a model built environment, the International Space Station, relative to human hosts and soil. mSystems.

[7] Lang JM, Coil DA, Neches RY, Brown WE, Cavalier D, Severance M (2017). A microbial survey of the International Space Station (ISS). PeerJ.

[8] Mora M, Wink L, Kögler I, Mahnert A, Rettberg P, Schwendner P (2019). Space Station conditions are selective but do not alter microbial characteristics relevant to human health. Nat Commun.

[9] Morris DW, Goldschmidt M, Keene H, Cron SG (2014). Microbial contamination of power toothbrushes: a comparison of solid-head versus hollow-head designs. J Dent Hyg..

[10] Karibasappa GN, Nagesh L, Sujatha BK (2011). Assessment of microbial contamination of toothbrush head: an in vitro study. Indian J Dent Res..

[11] Taji SS, Rogers AH (1998). The microbial contamination of toothbrushes. A pilot study. Aust Dent J..

[12] Warren DP, Goldschmidt MC, Thompson MB, Adler-Storthz K, Keene HJ (2001). The effects of toothpastes on the residual microbial contamination of toothbrushes. J Am Dent Assoc..

[13] Belda-Ferre P, Alcaraz LD, Cabrera-Rubio R, Romero H, Simón-Soro A, Pignatelli M (2012). The oral metagenome in health and disease. ISME J..

[14] Börnigen D, Ren B, Pickard R, Li J, Ozer E, Hartmann EM (2017). Alterations in oral bacterial communities are associated with risk factors for oral and oropharyngeal cancer. Sci Rep..

[15] Bik EM, Long CD, Armitage GC, Loomer P, Emerson J, Mongodin EF (2010). Bacterial diversity in the oral cavity of 10 healthy individuals. ISME J.

[16] Freire M, Moustafa A, Harkins DM, Torralba MG, Zhang Y, Leong P, et al. Longitudinal study of oral microbiome variation in twins. Sci Rep. 2020;10 Available from: http://www.nature.com/articles/s41598-020-64747-1. 10.1038/s41598-020-64747-1 PMC7224172 32409670

[17] Teng F, Yang F, Huang S, Bo C, Xu ZZ, Amir A (2015). Prediction of early childhood caries via spatial-temporal variations of oral microbiota. Cell Host Microbe.

[18] Johnson CR, Tran MN, Michelitsch LM, Abraham S, Hu J, Gray KA, et al. Nano-enabled, antimicrobial toothbrushes – how physical and chemical properties relate to antibacterial capabilities. J Hazard Mater. 2020;396. 10.1016/j.jhazmat.2020.122445. 10.1016/j.jhazmat.2020.122445 32298860

[19] do Nascimento C, Sorgini MB, Pita MS, Fernandes FHCN, Calefi PL, Watanabe E (2014). Effectiveness of three antimicrobial mouthrinses on the disinfection of toothbrushes stored in closed containers: a randomized clinical investigation by DNA checkerboard and culture. Gerodontology.

[20] Lloyd-Price J, Mahurkar A, Rahnavard G, Crabtree J, Orvis J, Hall AB (2017). Strains, functions and dynamics in the expanded Human Microbiome Project. Nature.

[21] Dias MF, da Rocha FG, Cristina de Paiva M, Christina de Matos Salim A, Santos AB, Amaral Nascimento AM. Exploring the resistome, virulome and microbiome of drinking water in environmental and clinical settings. Water Res. 2020;174:115630. 10.1016/j.watres.2020.115630. 10.1016/j.watres.2020.115630 32105997

[22] Ma L, Li B, Jiang XT, Wang YL, Xia Y, Li AD, et al. Catalogue of antibiotic resistome and host-tracking in drinking water deciphered by a large scale survey. Microbiome. 2017;5:154. https://microbiomejournal.biomedcentral.com/articles/10.1186/s40168-017-0369-0. 10.1186/s40168-017-0369-0 PMC5704573 29179769

[23] Knights D, Kuczynski J, Charlson ES, Zaneveld J, Mozer MC, Collman RG (2011). Bayesian community-wide culture-independent microbial source tracking. Nat Methods..

[24] Jia B, Raphenya AR, Alcock B, Waglechner N, Guo P, Tsang KK (2017). CARD 2017: Expansion and model-centric curation of the comprehensive antibiotic resistance database. Nucleic Acids Res..

[25] Mark Welch JL, Rossetti BJ, Rieken CW, Dewhirst FE, Borisy GG (2016). Biogeography of a human oral microbiome at the micron scale. Proc Natl Acad Sci..

[26] Chang YT, Lin CY, Chen YH, Hsueh PR. Update on infections caused by Stenotrophomonas maltophilia with particular attention to resistance mechanisms and therapeutic options. Front Microbiol. 2015;6:893. 10.3389/fmicb.2015.00893. 10.3389/fmicb.2015.00893 PMC4557615 26388847

[27] Coil DA, Neches RY, Lang JM, Brown WE, Severance M, Cavalier D (2016). Growth of 48 built environment bacterial isolates on board the International Space Station (ISS). PeerJ.

[28] Davin-Regli A, Pagès JM. Enterobacter aerogenes and Enterobacter cloacae; Versatile bacterial pathogens confronting antibiotic treatment. Front Microbiol. 2015;6:392. 10.3389/fmicb.2015.00392. 10.3389/fmicb.2015.00392 PMC4435039 26042091

[29] Emerson JB, Keady PB, Brewer TE, Clements N, Morgan EE, Awerbuch J (2015). Impacts of flood damage on airborne bacteria and fungi in homes after the 2013 Colorado front range flood. Environ Sci Technol..

[30] Khan R, Kong HG, Jung YH, Choi J, Baek KY, Hwang EC (2016). Triclosan resistome from metagenome reveals diverse enoyl acyl carrier protein reductases and selective enrichment of triclosan resistance genes. Sci Rep.

[31] Han J, Qiu W, Campbell EC, White JC, Xing B (2017). Nylon bristles and elastomers retain centigram levels of triclosan and other chemicals from toothpastes: accumulation and uncontrolled release. Environ Sci Technol..

[32] Abeles SR, Robles-Sikisaka R, Ly M, Lum AG, Salzman J, Boehm TK (2014). Human oral viruses are personal, persistent and gender-consistent. ISME J.

[33] Misic AM, Davis MF, Tyldsley AS, Hodkinson BP, Tolomeo P, Hu B, et al. The shared microbiota of humans and companion animals as evaluated from Staphylococcus carriage sites. Microbiome. 2015;3:2. https://microbiomejournal.biomedcentral.com/articles/10.1186/s40168-014-0052-7. 10.1186/s40168-014-0052-7 PMC4335418 25705378

[34] Patil S, Sanketh D, Amrutha N (2013). Oral microbial flora in health. World J Dent..

[35] Raju SC, Lagström S, Ellonen P, De Vos WM, Eriksson JG, Weiderpass E (2019). Gender-specific associations between saliva microbiota and body size. Front Microbiol..

[36] Veltri RW, Sprinkle PM, Keller SA, Chicklo JM (1972). Ecological alterations of oral microflora subsequent to tonsillectomy and adenoidectomy. J Laryngol Otol..

[37] Kembel SW, Jones E, Kline J, Northcutt D, Stenson J, Womack AM (2012). Architectural design influences the diversity and structure of the built environment microbiome. ISME J.

[38] Green JL (2014). Can bioinformed design promote healthy indoor ecosystems?. Indoor Air..

[39] Gibbons SM (2016). The built environment is a microbial wasteland. mSystems.

[40] Cardinale M, Kaiser D, Lueders T, Schnell S, Egert M (2017). Microbiome analysis and confocal microscopy of used kitchen sponges reveal massive colonization by Acinetobacter, Moraxella and Chryseobacterium species. Sci Rep.

[41] Raghupathi PK, Zupančič J, Brejnrod AD, Jacquiod S, Houf K, Burmølle M (2018). Microbial diversity and putative opportunistic pathogens in dishwasher biofilm communities. Appl Environ Microbiol..

[42] Schmithausen RM, Sib E, Exner M, Hack S, Rösing C, Ciorba P (2019). The washing machine as a reservoir for transmission of extended-spectrum-beta-lactamase (CTX-M-15)-producing Klebsiella oxytoca ST201 to newborns. Appl Environ Microbiol..

[43] Stephens B (2016). What have we learned about the microbiomes of indoor environments?. mSystems.

[44] Hu J, Ben Maamar S, Glawe AJ, Gottel N, Gilbert JA, Hartmann EM (2019). Impacts of indoor surface finishes on bacterial viability. Indoor Air..

[45] Rodriguez-R LM, Gunturu S, Tiedje JM, Cole JR, Konstantinidis KT (2018). Nonpareil 3: fast estimation of metagenomic coverage and sequence diversity. mSystems.

[46] Truong DT, Franzosa EA, Tickle TL, Scholz M, Weingart G, Pasolli E (2015). MetaPhlAn2 for enhanced metagenomic taxonomic profiling. Nat Methods..

[47] Kaminski J, Gibson MK, Franzosa EA, Segata N, Dantas G, Huttenhower C (2012). Structure, function and diversity of the healthy human microbiome. Nature..

[48] Suzek BE, Wang Y, Huang H, McGarvey PB, Wu CH (2015). UniRef clusters: a comprehensive and scalable alternative for improving sequence similarity searches. Bioinformatics..

[49] Arkin AP, Cottingham RW, Henry CS, Harris NL, Stevens RL, Maslov S (2018). KBase: The United States department of energy systems biology knowledgebase. Nat Biotechnol..

[50] Bolger AM, Lohse M, Usadel B (2014). Trimmomatic: a flexible trimmer for Illumina sequence data. Bioinformatics..

[51] Li D, Liu CM, Luo R, Sadakane K, Lam TW (2015). MEGAHIT: an ultra-fast single-node solution for large and complex metagenomics assembly via succinct de Bruijn graph. Bioinformatics..

[52] Wu Y, Simmons BA, Singer SW (2016). MaxBin 2.0: an automated binning algorithm to recover genomes from multiple metagenomic datasets. Bioinformatics.

[53] Parks DH, Imelfort M, Skennerton CT, Hugenholtz P, Tyson GW (2015). CheckM: assessing the quality of microbial genomes recovered from. Cold Spring Harb Lab Press Method..

[54] Dixon P (2003). Computer program review VEGAN, a package of R functions for community ecology. J Veg Sci.

