# Supplementary figures and images for: Toothbrush microbiomes feature a meeting ground for human oral and environmental microbiota
Source: Microbiome. 2021 Jan 31;9:32. doi: 10.1186/s40168-020-00983-x (PMC7849112; doi:10.1186/s40168-020-00983-x)

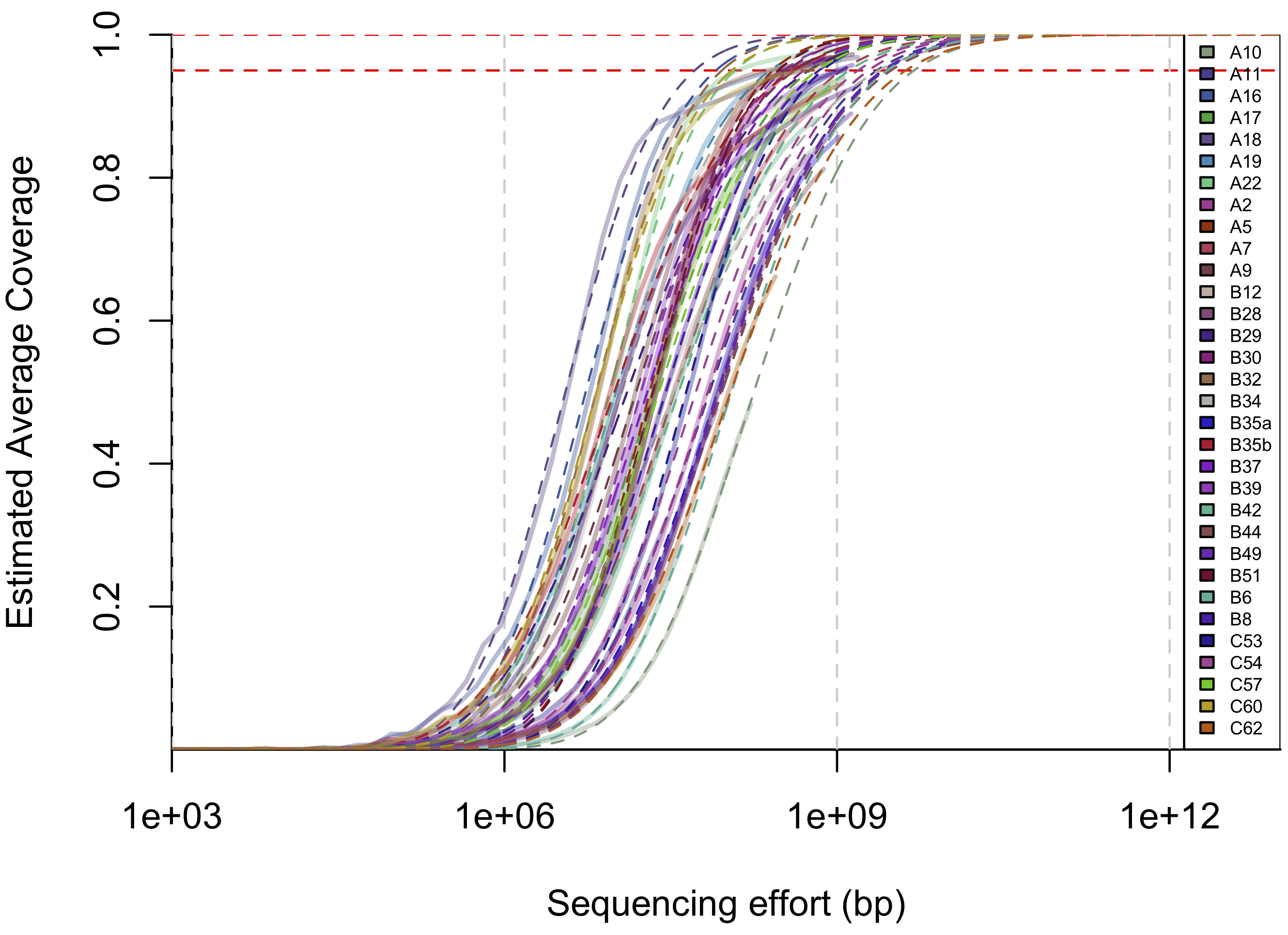

Supplement: Supplementary file 2 — Additional file 1: Figure S1. Nonpareil curves illustrating sequencing coverage of the toothbrush metagenomes. Solid and dotted lines correspond to observed and predicted redundancy, respectively. [file 40168_2020_983_MOESM2_ESM.png]

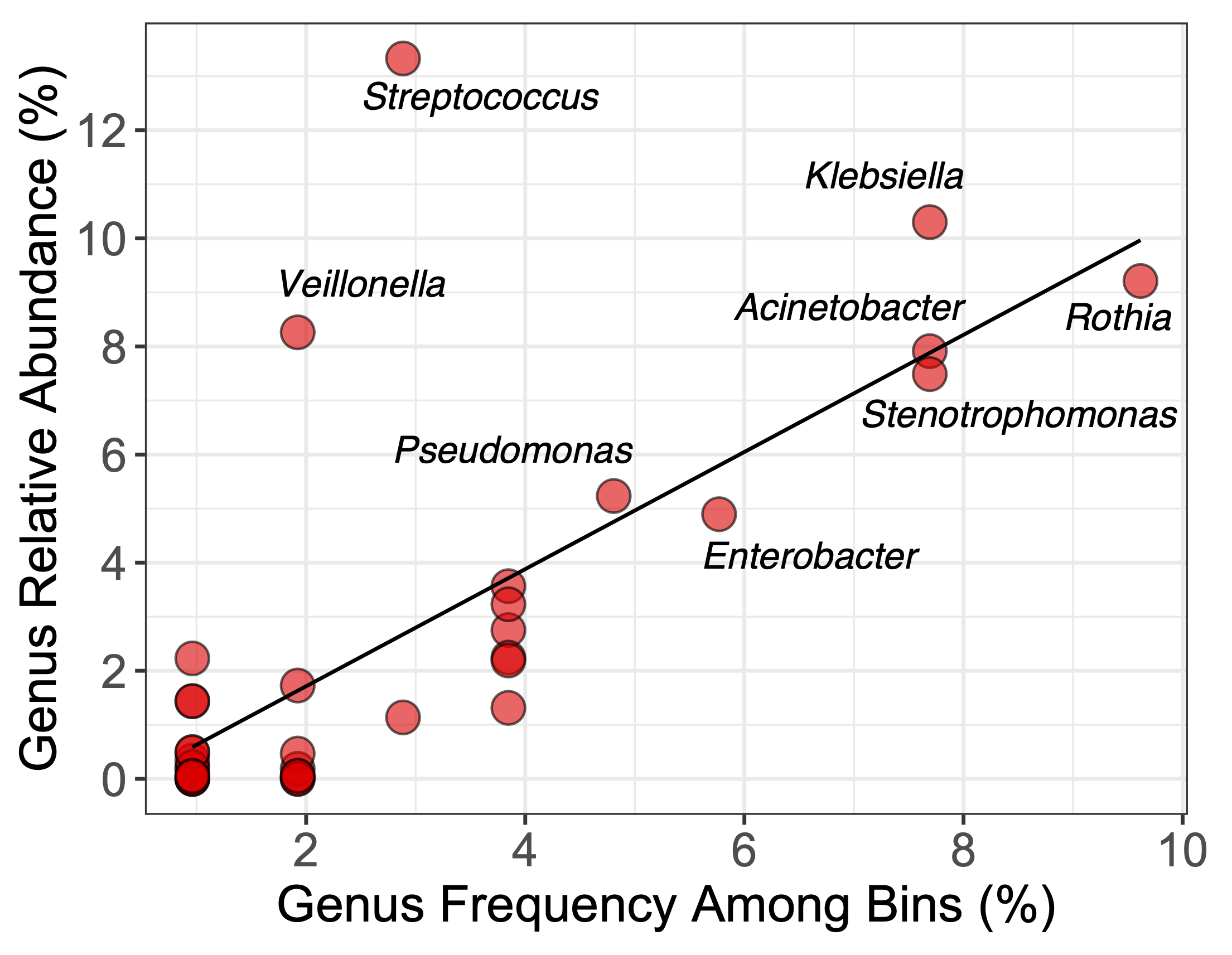

Supplement: Supplementary file 5 — Additional file 4: Figure S2. Relationship between genera relative abundances within toothbrush microbial communities predicted using the marker-gene approach (i.e., MetaPhlAn2) and genera frequencies among metagenome-assembled genomes. Genera with over 5% from either category are listed. [file 40168_2020_983_MOESM5_ESM.png]

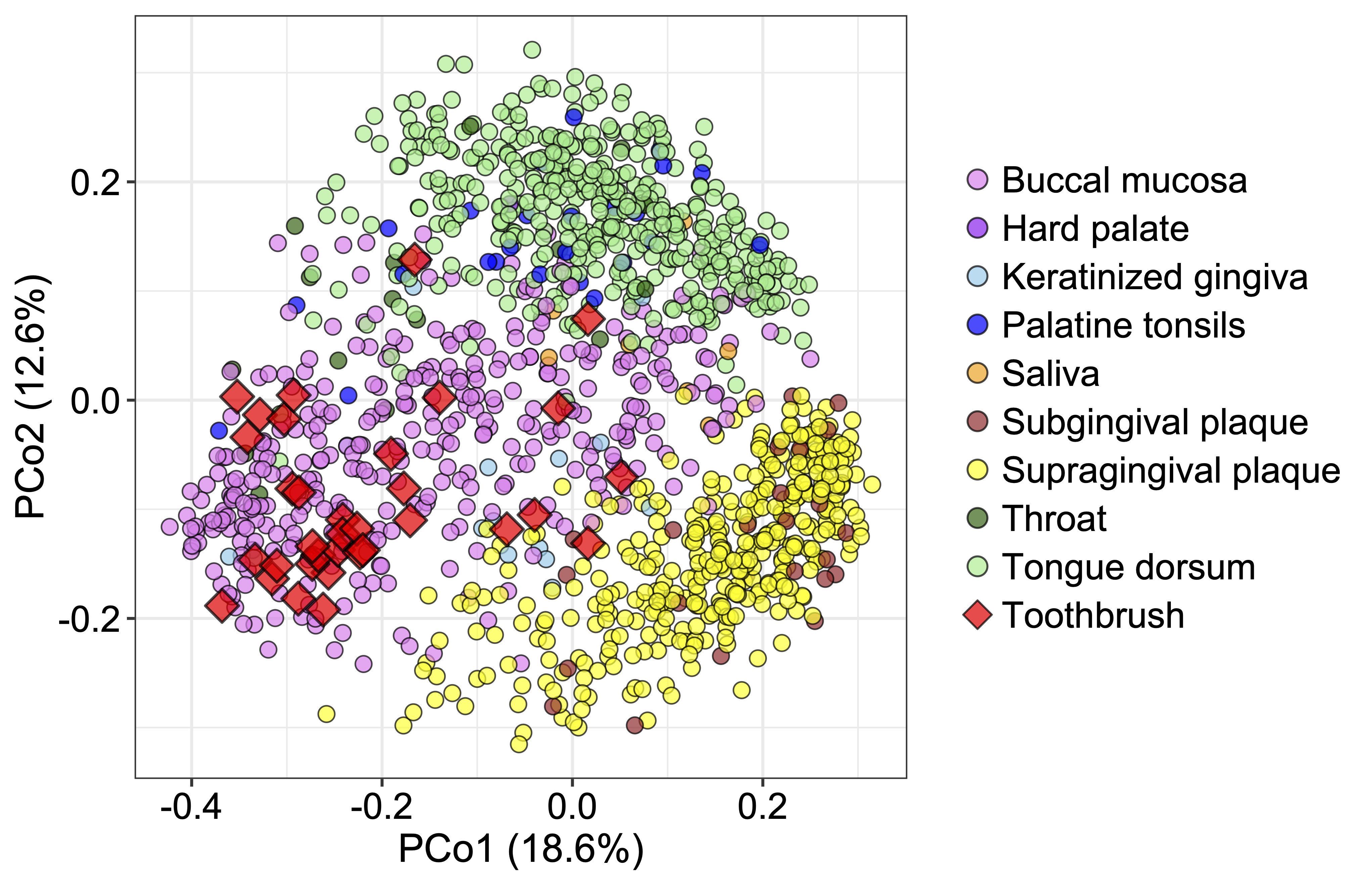

Supplement: Supplementary file 6 — Additional file 5: Figure S3. PCoA displaying species-level beta-diversity across microbial communities on toothbrushes and those from specific oral sites (HMP-II). Colors/shapes corresponds to sample type. [file 40168_2020_983_MOESM6_ESM.png]

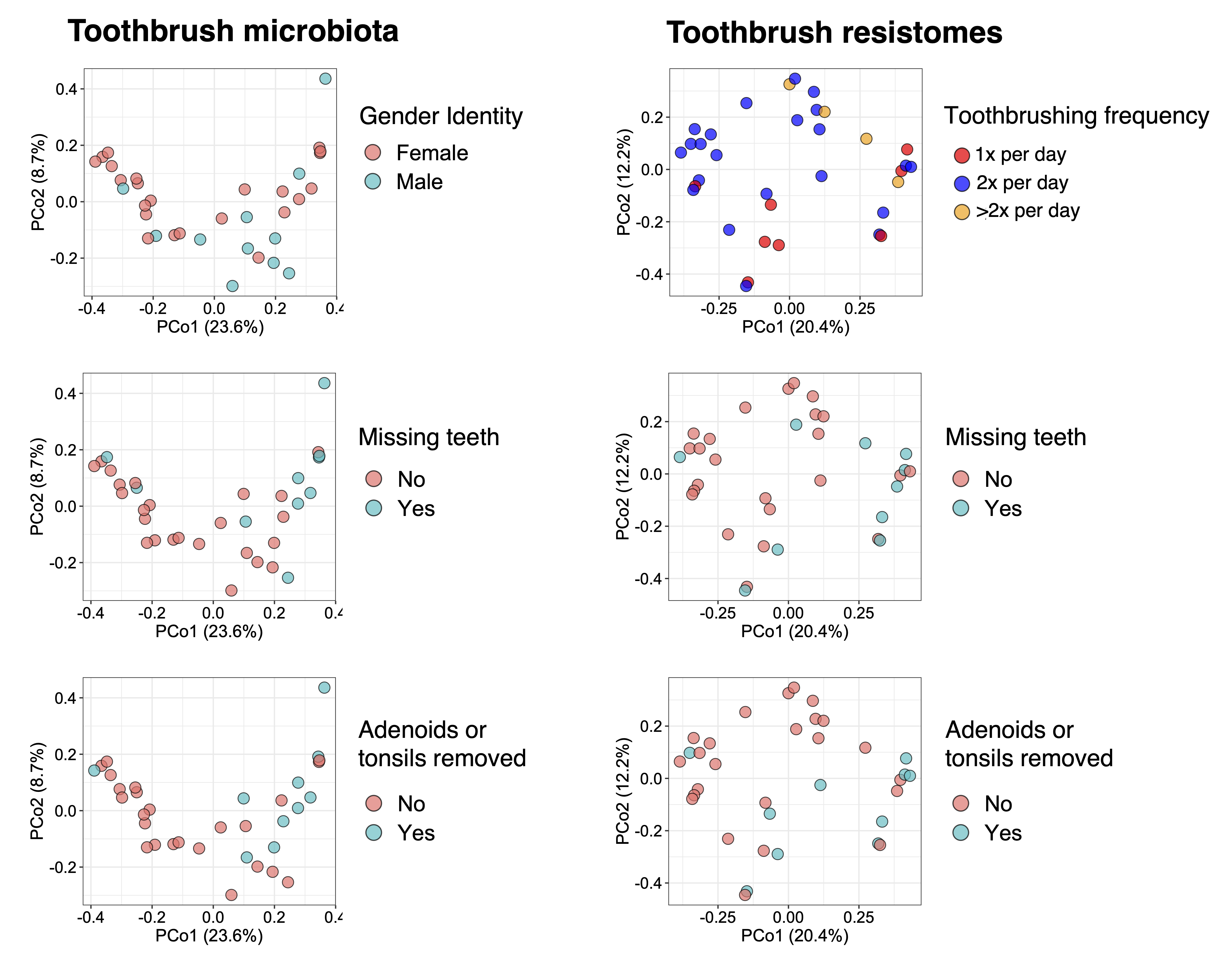

Supplement: Supplementary file 9 — Additional file 8: Figure S4. Metadata that may associate with beta-diversity of toothbrush microbiota (left panels) and toothbrush resistomes (right panels), i.e., factors from the set of 20 variables displayed in Figure 4 that had a PERMANOVA p < 0.1. [file 40168_2020_983_MOESM9_ESM.png]

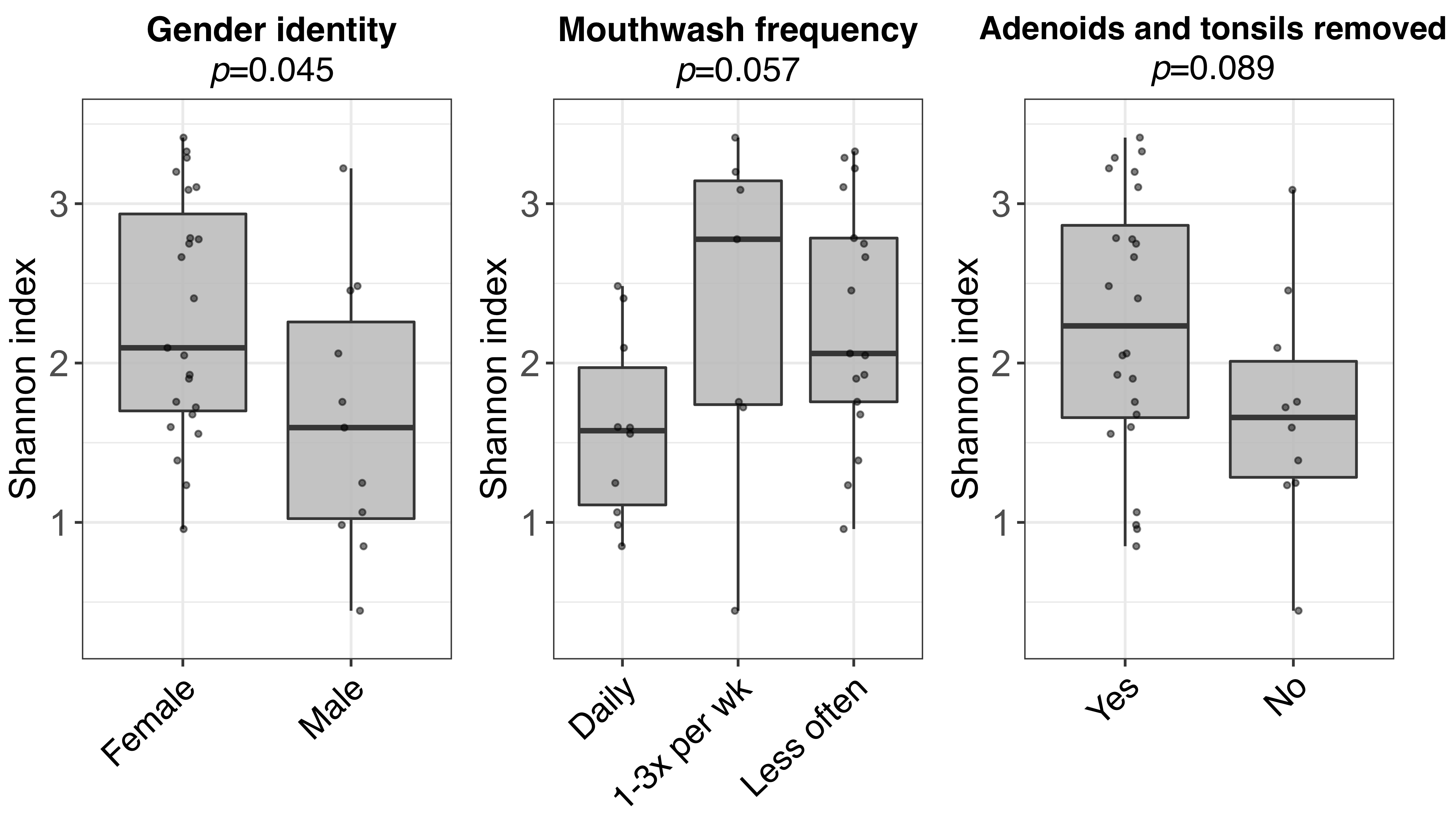

Supplement: Supplementary file 10 — Additional file 9: Figure S5. Metadata that may associate with alpha-diversity of toothbrush microbiota taxonomic profiles; i.e., factors from the set of 20 variables displayed in Figure 4 that had a Mann-Whitney or Kruskal-Wallis test p < 0.1. [file 40168_2020_983_MOESM10_ESM.png]

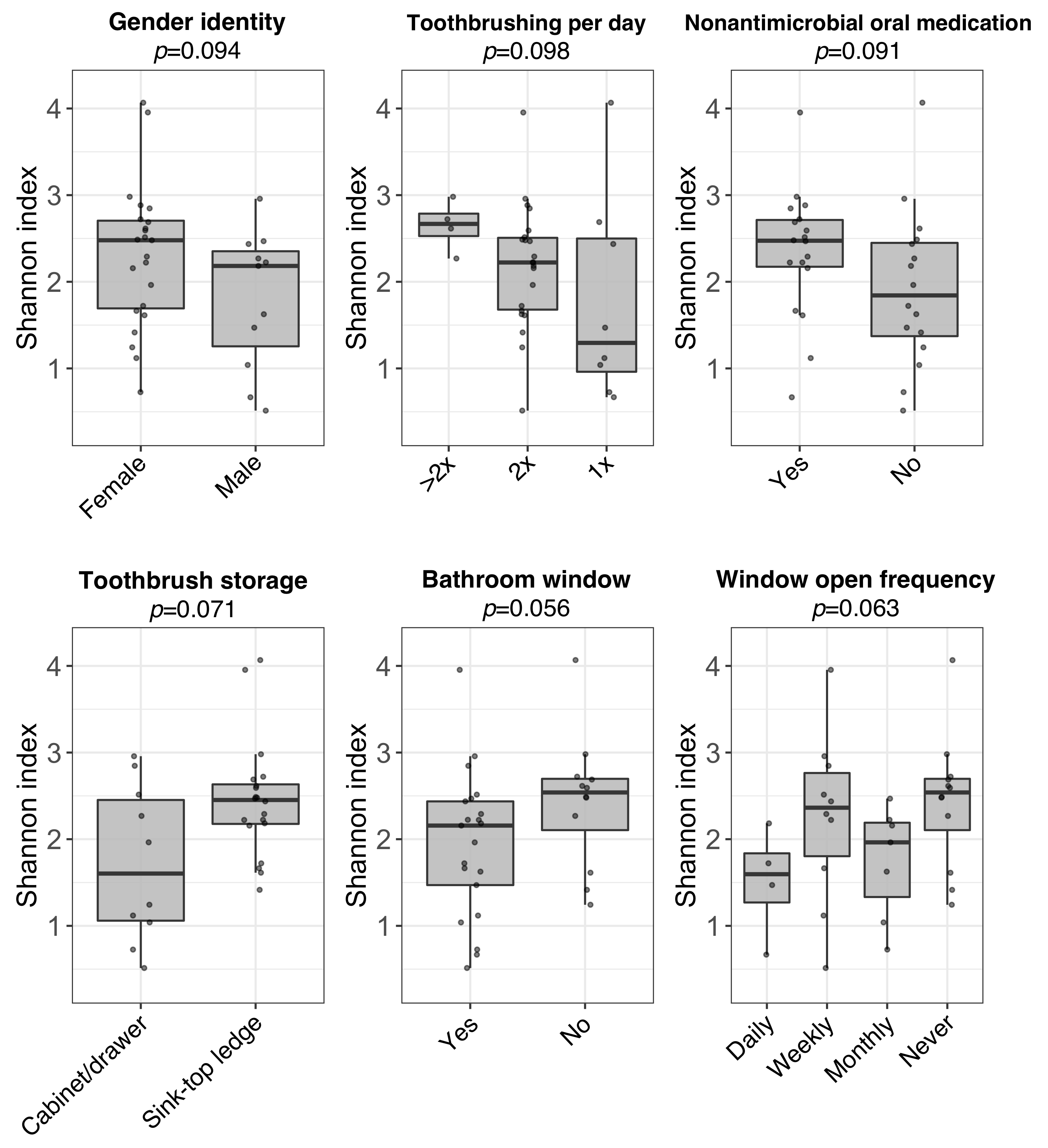

Supplement: Supplementary file 11 — Additional file 10: Figure S6. Metadata that may associate with alpha-diversity of toothbrush resistomes, i.e., factors from the set of 20 variables displayed in Figure 4 that had a Mann-Whitney or Kruskal-Wallis test p < 0.1. [file 40168_2020_983_MOESM11_ESM.png]
