# Supplementary material for: Toothbrush microbiomes feature a meeting ground for human oral and environmental microbiota
Source: Microbiome. 2021 Jan 31;9:32. doi: 10.1186/s40168-020-00983-x (PMC7849112; doi:10.1186/s40168-020-00983-x)
Supplement: Supplementary file 12 — Additional file 11: Dat. S1. Sample survey questionnaire that was used to collect metadata. [file 40168_2020_983_MOESM12_ESM.pdf]

TMP Participant Survey

Please complete the survey below.

Thank you!

Welcome to the Toothbrush Microbiome Project participant survey! Your personal information will be used for research purposes in this study only. After completion, we will mail you a TMP kit with instructions to provide your toothbrush, saliva, and cheek swab samples. If you have any questions, please contact Prof. Erica Hartmann at erica.hartmann@northwestern.edu.

Please read the attached document and proceed with electronic signature.

[Attachment: "HRP-592_biomedical_consent_final.pdf"]

1) The researcher may contact me in the future to see whether I am interested in participating in other research studies by the Principal Investigator of this study.
   ◯ I agree
   ◯ I disagree

2) The researcher may retain any leftover tissue (cheek swab) samples taken during the study. These samples may be used for other research not related to this study. These samples will be retained in non-identifiable form, meaning that there will be no information associated with the blood or samples that will allow anyone to readily ascertain my identity.
   ◯ I agree
   ◯ I disagree

3) Your signature documents your permission to take part in this research. You will be provided a copy of this signed document.
   ____

4) Mailing address. We will need this information to ship your TMP kit (it will contain return shipping materials/instructions)
   ____

5) Phone number (NA if want to remain anonymous)
   ____

6) Email address (NA if want to remain anonymous)
   ____

Personal/Health Information

7) Gender
- ◯ Male
- ◯ Female
- ◯ Do not wish to disclose

8) Age ________________________

9) How many cavities have you had in your adult teeth?
- ◯ 0
- ◯ 1-5
- ◯ 5-10
- ◯ 10+
- ◯ Unsure

10) Have you ever had or do you currently have oral infection?
- ☐ None apply
- ☐ Gingivitis
- ☐ Periodontitis
- ☐ Other

11) If "other", please indicate ________________________

12) Select any current dental devices
- ☐ None apply
- ☐ Braces
- ☐ Retainer
- ☐ Night guard
- ☐ Other

13) If "other", please indicate ________________________

14) Are you currently on, or have you in the past two weeks, taken any antibiotics?
- ◯ Yes
- ◯ No

15) If "yes", please indicate (not required) ________________________

16) Are you currently taking any other oral medications?
- ◯ Yes
- ◯ No

17) If "yes", please indicate (not required) ________________________

18) How many teeth are you missing?
- ◯ 0
- ◯ 1-2
- ◯ 3-5
- ◯ 5+

19) Have you had any teeth removed (including "wisdom teeth")?
- ◯ Yes
- ◯ No

20) Have you had your adenoids and/or tonsils removed?
- ◯ Yes
- ◯ No

21) Do you use dentures?
- ◯ Yes
- ◯ No

22) Do you have any tooth implants?
- ◯ Yes
- ◯ No

23) Do you have any tongue/mouth piercings?
- ◯ Yes
- ◯ No

24) Do you use any of the following tobacco products?

- ☐ None
- ☐ Cigarettes
- ☐ Cigars
- ☐ Chew
- ☐ Vaporizer

25) Do you regularly use marijuana (including medical or recreational)?

- ○ Yes
- ○ No

26) About how many alcohol beverages do you consume per week?

- ○ 0
- ○ 1-5
- ○ 5-10
- ○ 10-20
- ○ 20+

27) How often do you consume coffee?

- ○ daily
- ○ few times per week
- ○ few times per month
- ○ never

28) Please select any of the following dietary restrictions

- ☐ None apply
- ☐ Vegan
- ☐ Vegetarian
- ☐ Pescatarian
- ☐ Other

29) Do you have any pets? Check all that apply

- ☐ No pets
- ☐ Yes, Dog
- ☐ Yes, Cat
- ☐ Yes, Other

Oral Care Information

30) What is your current toothpaste brand?
- ◯ Colgate
- ◯ Aquafresh
- ◯ Crest
- ◯ Other

31) If "other", please indicate __________________________________

32) Is it an antimicrobial toothpaste?
- ◯ Yes
- ◯ No
- ◯ Unsure

33) Please specify ANY/ALL active ingredients listed on the toothpaste package __________________________________

34) What is your current toothbrush brand? __________________________________

35) Is it an antimicrobial toothbrush?
- ◯ Yes
- ◯ No
- ◯ Unsure

36) If "yes", please list the antimicrobial agent if known __________________________________

37) For what duration of time have you been using your current toothbrush?
- ◯ < 2 weeks
- ◯ 2 weeks - 1 month
- ◯ 1 - 2 months
- ◯ 2 - 3 months
- ◯ 3 - 4 months
- ◯ > 4 months

38) On average, how often do you brush your teeth per day?
- ◯ 1x
- ◯ 2x
- ◯ 3x
- ◯ >3x

39) How would you best describe storage of your toothbrush?
- ☐ Sink ledge
- ☐ Cabinet or drawer
- ☐ Other

40) Do you use a toothbrush head cover?
- ◯ Yes
- ◯ No

41) Is your toothbrush stored in close proximity to another toothbrush?
- ◯ Yes
- ◯ No

42) If "yes", who does the other toothbrush belong to?
- ◯ Partner
- ◯ Roommate

43) Do you use any disinfecting techniques for your toothbrush (e.g., boiling water, UV)?
- ◯ Yes
- ◯ No

44) How often do you use dental floss?
- ◯ 1x per day
- ◯ 2-3x per week
- ◯ 1x per week
- ◯ 1x per month
- ◯ < 1x per month

45) How often do you use mouthwash?

- ◯ 1x per day
- ◯ 2-3x per week
- ◯ 1x per week
- ◯ 1 x per month
- ◯ < 1x per month

46) If you use mouthwash, please list the brand and specify any active ingredients listed on the package

____

Features in Bathroom (or room where toothbrush is stored)

47) Please provide a rough approximation of the dimensions of the bathroom in LxWxH (e.g., 5ft x 5ft x 10ft) ______________________

48) Approximately how many feet from the toilet is your toothbrush being stored (if it is in the bathroom)? ______________________

49) Is there a shower in the bathroom?
- ◯ Yes
- ◯ No

50) Is there a window in the bathroom?
- ◯ Yes
- ◯ No

51) If "yes", please estimate window area in W x H (e.g., 2ft x 3ft) ______________________

52) If "yes", how often is the window opened?
- ◯ daily
- ◯ weekly
- ◯ monthly
- ◯

53) Is there an HVAC (i.e., central air/heat) system in the house/apartment?
- ◯ Yes
- ◯ No
- ◯ Unsure

54) Are you using a water filter in the sink that is used when brushing your teeth?
- ◯ Yes
- ◯ No

Follow-up

55) Would you be willing to participate in a follow-up study for toothbrush microbiome monitoring over time?
◯ Yes
◯ No

56) Do you grant permission for any bacteria cultures from your toothbrush sample to be used in future research?
◯ Yes
◯ No

57) Would you like us to send you results from your toothbrush and/or oral microbiome/resistome (via email)?
◯ Yes
◯ No
